# A new tetra­kis-substituted pyrazine carb­oxy­lic acid, 3,3′,3′′,3′′′-{[pyrazine-2,3,5,6-tetra­yltetra­kis(methyl­ene)]tetra­kis­(sulfanedi­yl)}tetra­propionic acid: crystal structures of two triclinic polymorphs and of two potassium–organic frameworks

**DOI:** 10.1107/S2056989021003479

**Published:** 2021-04-09

**Authors:** Jessica Pacifico, Helen Stoeckli-Evans

**Affiliations:** a Institute of Chemistry, University of Neuchâtel, Av. de Bellevaux 51, CH-2000 Neuchâtel, Switzerland; b Institute of Physics, University of Neuchâtel, rue Emile-Argand 11, CH-2000 Neuchâtel, Switzerland

**Keywords:** crystal structure, pyrazine, tetra­kis­, carboxyl­ate, polymorphism, hydrogen bonding, supra­molecular framework, alkali metal, potassium-organic framework, Hirshfeld surface, energy framework

## Abstract

Two polymorphs of the title tetra­kis-substituted pyrazine carb­oxy­lic acid, 3,3′,3′′,3′′′-{[pyrazine-2,3,5,6-tetra­yltetra­kis­(methyl­ene))tetra­kis­(sulfanedi­yl]}tetra­propionic acid, C_20_H_28_N_2_O_8_S_4_, (**H_4_L1**), have been obtained, **H_4_L1_A** and **H_4_L1_B**. Each structure crystallized with half a mol­ecule in the asymmetric unit of a triclinic *P*  unit cell. The whole mol­ecules are generated by inversion symmetry, with the pyrazine rings being located about inversion centers. The crystals of **H_4_L1_B** were of poor quality, but the X-ray diffraction analysis does show the change in conformation of the –CH_2_—S—CH_2_—CH_2_– side chains compared to those in polymorph **H_4_L1_A**. In the crystal of **H_4_L1_A**, mol­ecules are linked by two pairs of O—H⋯O hydrogen bonds, enclosing *R* ^2^_2_(8) ring motifs forming layers parallel to plane (100), which are linked by C—H⋯O hydrogen bonds to form a supra­molecular framework. In the crystal of **H_4_L1_B**, mol­ecules are also linked by two pairs of O—H⋯O hydrogen bonds enclosing *R* ^2^_2_(8) ring motifs, however here, chains are formed propagating in the [001] direction and stacking up the *a*-axis. Reaction of **H_4_L1** with Hg(NO_3_)_2_ in the presence of a potassium acetate buffer did not produce the expected binuclear complex, instead crystals of a potassium–organic framework were obtained, poly[(μ-3-{[(3,5,6-tris­{[(2-carb­oxy­eth­yl)sulfan­yl]meth­yl}pyrazin-2-yl)meth­yl]sulfan­yl}propano­ato)potassium], [K(C_20_H_27_N_2_O_8_S_4_)]_*n*_ (**KH_3_L1**). The organic mono-anion possesses inversion symmetry with the pyrazine ring being located about an inversion center. A carb­oxy H atom is disordered by symmetry and the charge is compensated for by a potassium ion. A similar reaction with Zn(NO_3_)_2_ resulted in the formation of crystals of a dipotassium-organic framework, poly[(μ-3,3′-{[(3,6-bis­{[(2-carb­oxy­eth­yl)sulfan­yl]meth­yl}pyrazine-2,5-di­yl)bis(methyl­ene)]bis­(sulfanedi­yl)}dipropionato)dipotassium], [K_2_(C_20_H_26_N_2_O_8_S_4_)]_*n*_ (**K_2_H_2_L1**). Here, the organic di-anion possesses inversion symmetry with the pyrazine ring being located about an inversion center. Two symmetry-related acid groups are deprotonated and the charges are compensated for by two potassium ions.

## Abstract 2

The crystal structures of two triclinic polymorphs of a new tetra­kis-substituted pyrazine carb­oxy­lic acid, 3,3′,3′′,3′′′-[(pyrazine-2,3,5,6-tetra­yltetra­kis­(methyl­ene))tetra­kis­(sulfanedi­yl)]tetra­propionic acid, are reported, together with the crystal structures of two potassium-organic frameworks.

## Chemical context   

**Figure 1:**
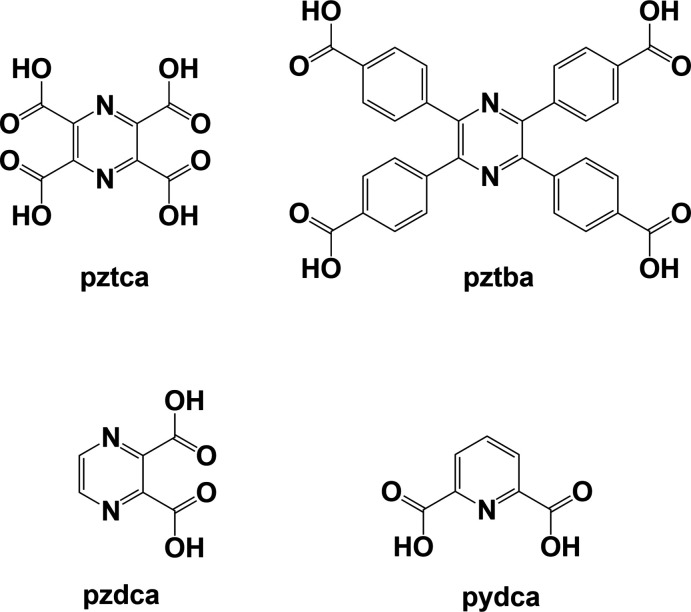
Chemical diagrams for pyrazine-2,3,5,6-tetra­carb­oxy­lic acid (**pztca**), 4,4′,4′′,4′′′-(pyrazine-2,3,5,6-tetra­yl)tetra­benzoic acid (**pztba**), pyrazine-2,3-di­carb­oxy­lic acid (**pzdca**) and pyridine-2,6-di­carb­oxy­lic acid (**pydca**).

The title tetrakis-substituted pyrazine carb­oxy­lic acid, 3,3′,3′′,3′′′-[(pyrazine-2,3,5,6-tetra­yltetra­kis­(methyl­ene))tetra­kis­(sulfane­di­yl)]tetra­propionic acid (**H_4_L1**), is to the best of our knowledge, only the third pyrazine tetrakis-substituted carb­oxy­lic acid ligand to have been synthesized. The first is pyrazine-2,3,5,6-tetra­carb­oxy­lic acid (**pztca**), which was originally synthesized by Wolff at the end of the 19th century (Wolff, 1887 ▸, 1893 ▸), while the second is 4,4′,4′′,4′′′-(pyrazine-2,3,5,6-tetra­yl)tetra­benzoic acid (**pztba**), which was first synthesized by Jiang *et al.* (2017 ▸). **Pztca** (Fig. 1 ▸) has been used to synthesize a number of coordination polymers, the first being poly{[(2,5-di­carb­oxy­pyrazine-3,6-di­carboxyl­ato)transdi­aqua­iron(II) dihydrate]} (Marioni *et al.*, 1986 ▸), while **pztba** (Fig. 1 ▸) has been shown to form a series of metal–organic frameworks (Jiang *et al.*, 2017 ▸; Wang *et al.*, 2019 ▸).
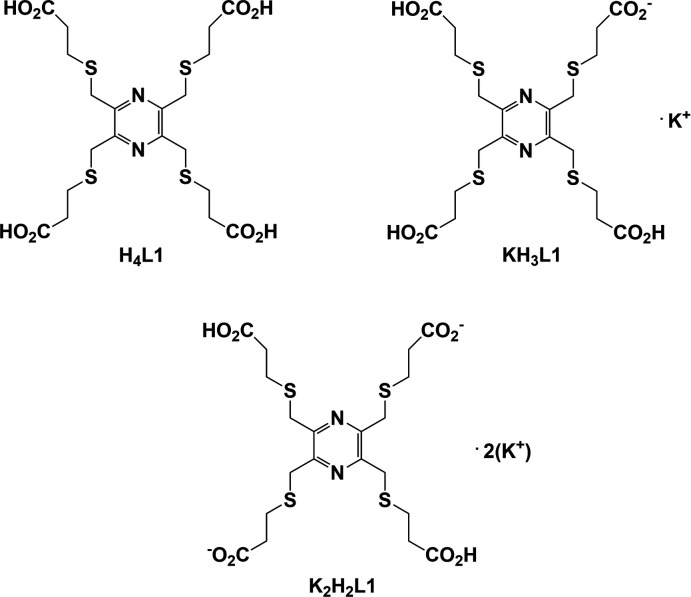


**Figure 2:**
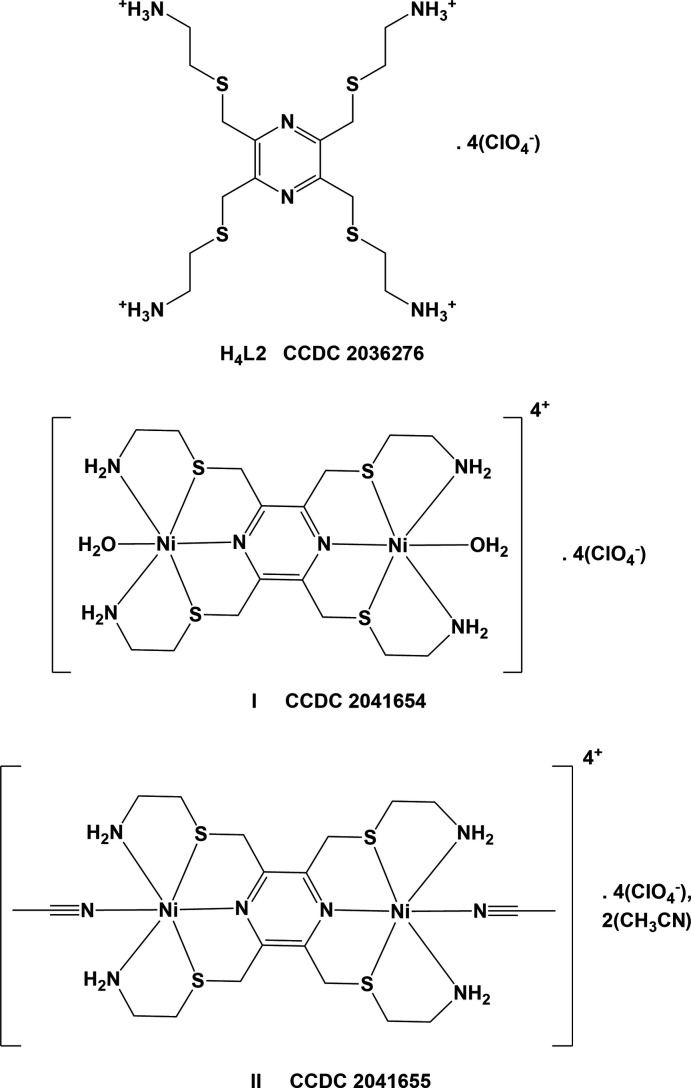
Chemical diagram for 2,2′,2′′,2′′′-[(pyrazine-2,3,5,6-tetra­yltetra­kis(methyl­ene)tetra­kis­(sulfanedi­yl)]tetra­kis­(ethan-1-amine) (**H_4_L2**) and two nickel(II) binuclear complexes, **I** and **II** (Pacifico & Stoeckli-Evans, 2020 ▸).

The title ligand was synthesized to study its coordination behaviour with various transition metal ions (Pacifico, 2003 ▸). Potentially the ligand can coordinate in a bis-penta­dentate manner, as was shown to be the case for a similar ligand, 2,2′,2′′,2′′′-{[pyrazine-2,3,5,6-tetra­yltetra­kis­(methyl­ene)]tetra­kis­(sulfanedi­yl)}tetra­kis­(ethan-1-amine) (**H_4_L2**), for which two nickel(II) binuclear complexes, **I** and **II**, were synthesized (Pacifico, 2003 ▸; Pacifico & Stoeckli-Evans, 2020 ▸); see Fig. 2 ▸.

## Structural commentary   

**Figure 3:**
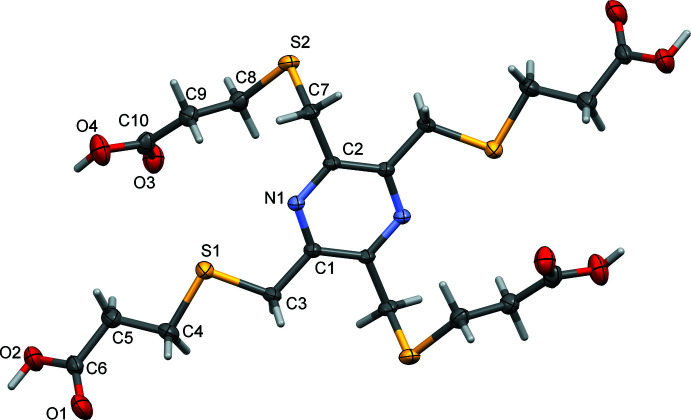
The mol­ecular structure of **H_4_L1_A**, with atom labelling. Displacement ellipsoids are drawn at the 50% probability level. Unlabelled atoms are related to labelled atoms by symmetry operator −*x* + 2, −*y* + 1, −*z* + 1.

The title tetra­kis-substituted pyrazine carb­oxy­lic acid, 3,3′,3′′,3′′′-[(pyrazine-2,3,5,6-tetra­yltetra­kis­(methyl­ene))tetra­kis­(sulfanedi­yl)]tetra­propionic acid (**H_4_L1_A**), crystallized with half a mol­ecule in the asymmetric unit (Fig. 3 ▸). The whole mol­ecule is generated by inversion symmetry, with the pyrazine ring being located about an inversion center.

**Figure 4:**
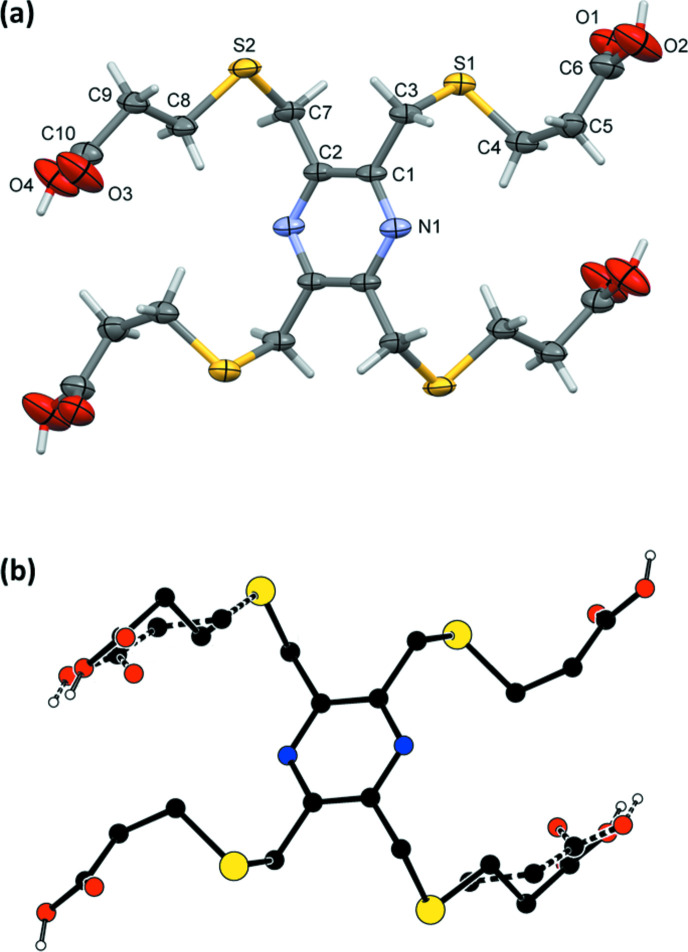
(*a*) The mol­ecular structure of **H_4_L1_B**, with atom labelling. Displacement ellipsoids are drawn at the 30% probability level. (*b*) A view of the mol­ecular structure of **H_4_L1_B** with the symmetry-related disordered side chains (C8/C8*B*, C9/C9*B*, C10/C10*B*, O3/O3*B* and O4/O4*B*) shown with dashed bonds. Unlabelled atoms are related to labelled atoms by symmetry operator −*x* + 2, −*y* + 1, −*z* + 1.

**Figure 5:**
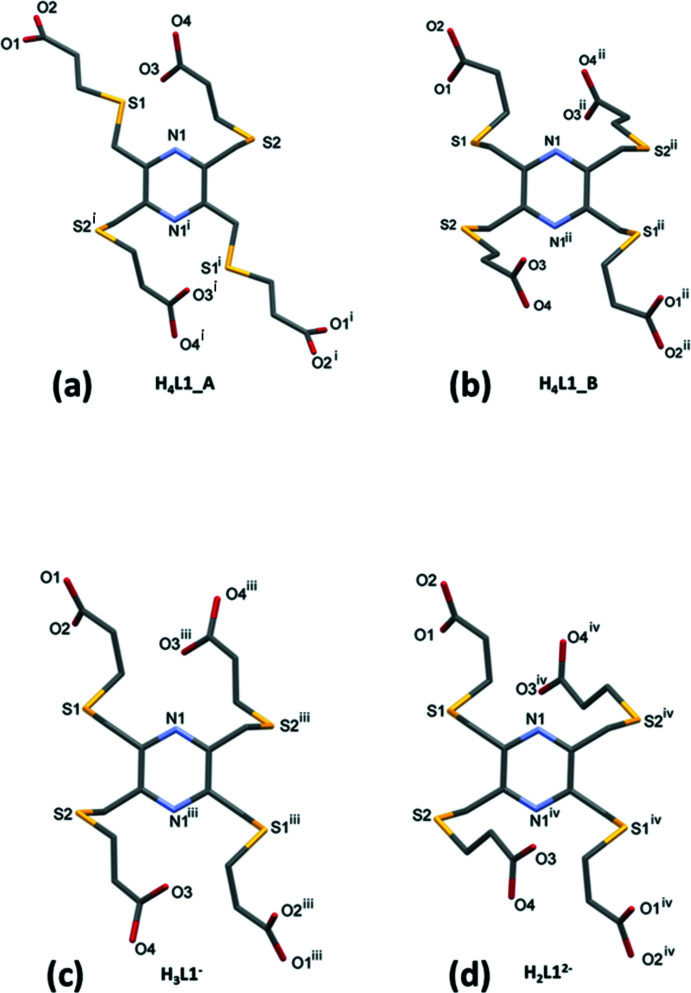
A comparison of the orientation of the –CH_2_—S—CH_2_—CH_2_– side chains in (*a*) polymorph **H_4_L1_A**, (*b*) for the major disordered component of polymorph **H_4_L1_B**, (*c*) **KH_3_L1** and (*d*) **K_2_H_2_L1** [see Table 1 ▸ for further details; symmetry codes: (i) = (ii) −*x* + 2, −*y* + 1, −*z* + 1; (iii) −*x* + 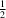, −*y* + 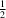, −*z*; (iv) −*x* + 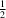, −*y* + 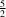, −*z* + 1].

In an attempt to form a co-crystal, equimolar amounts of **H_4_L1** and terephthalic acid were mixed in methanol. On slow evaporation of the solvent, colourless plate-like crystals were obtained. X-ray diffraction analysis revealed their structure to be that of a second triclinic *P* 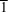 polymorph, **H_4_L1_B** (Fig. 4 ▸). It crystallized with half a mol­ecule in the asymmetric unit and the whole mol­ecule is generated by inversion symmetry, with the pyrazine ring being located about an inversion center. The crystals were of poor quality with one CH_2_—CH_2_—CO_2_H side chain (atoms C8/C8*B*, C9/C9*B*, C10/C10*B*, O3/O3*B*, O4/O4*B*) of the centrosymmetric mol­ecule being positionally disordered (Fig. 4 ▸ *b*). The difference in the two polymorphs is essentially in the orientation of the –CH_2_—S—CH_2_—CH_2_—C– side arms, as shown in Fig. 5 ▸ *a* and *b*. Selected torsion angles are given in Table 1 ▸.

**Figure 6:**
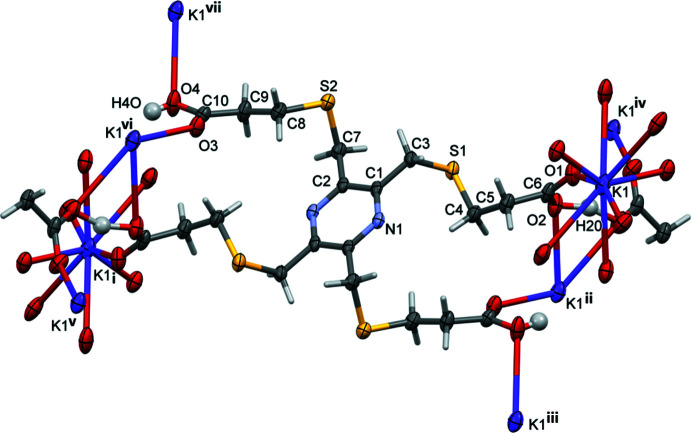
The mol­ecular structure of complex **KH_3_L1**, with labels for the atoms in the asymmetric unit of the organic anion. Unlabelled atoms are related to labelled atoms by symmetry operator (i) −*x* + 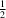, −*y* + 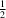, −*z*. Displacement ellipsoids are drawn at the 50% probability level. [Further symmetry codes are: (ii) −*x* + 1, −*y*, −*z*: (iii) *x*, *y* + 1, *z*; (iv) *x*, *y*, *z* + 1; (v) −*x* + 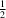, −*y* + 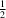, −*z* − 1; (vi) *x* − 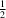, *y* + 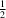, *z*; (vii) −*x* + 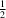, −*y* − 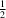, −*z*.]

**Figure 7:**
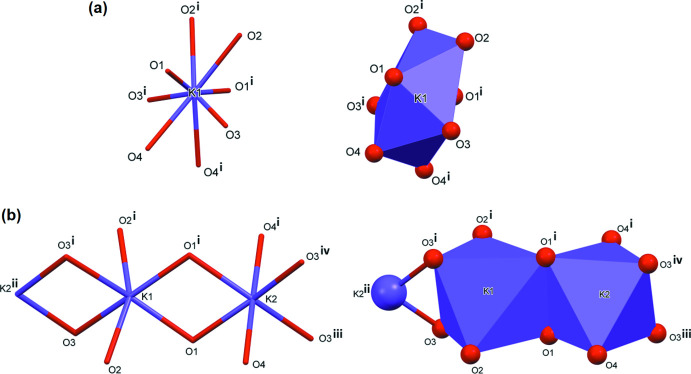
(*a*) Views of the coordination sphere of the potassium ion in **KH_3_L1** [symmetry code: (i) −*x* + 1, *y*, −*z* − 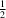] and (*b*) views of the coordination sphere of the potassium ions in **K_2_H_2_L1** [symmetry codes: (i) −*x*, *y*, −*z* + 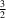; (ii) *x*, *y* − 1, *z*; (iii) *x*, *y* + 1, *z*; (iv) −*x*, *y* + 1, −*z* + 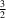].

Reaction of **H_4_L1** with Hg(NO_3_)_2_ in the presence a 1 *M* potassium acetate buffer led to the formation of colourless crystals that proved to be a potassium–organic framework (**KH_3_L1**); see Fig. 6 ▸. The asymmetric unit consists of half a mono-deprotonated ligand mol­ecule located about an inversion center, and half a potassium ion located on an inversion center. The carb­oxy H atom is disordered by symmetry. The K^+^ ion is linked to the O atoms of the acid groups and has a coordination number of eight (KO_8_) and a distorted dodeca­hedral geometry (Fig. 7 ▸ *a*). The K⋯O bond lengths vary between 2.682 (2) and 3.069 (3) Å (Table 2 ▸). Inter­estingly, here there is a significant difference between the K⋯O(C=O) and K⋯O(O^−^) distances: 2.6823 (2) and 2.828 (2) Å compared to 3.056 (3) and 3.069 (3) Å, respectively.

**Figure 8:**
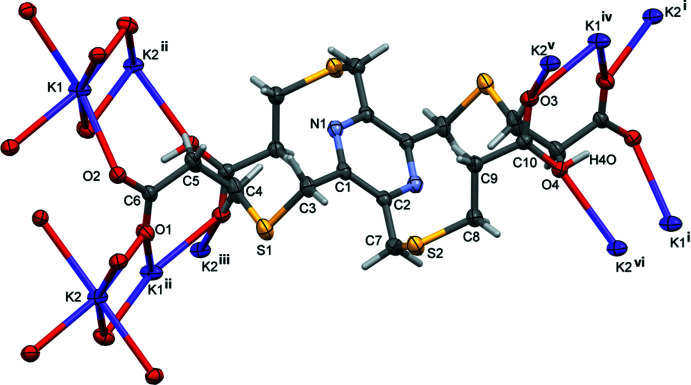
The mol­ecular structure of complex **K_2_H_2_L1**, with labels for the atoms in the asymmetric unit of the organic dianion. Unlabelled atoms are related to labelled atoms by symmetry operator (i) −*x* + 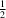, −*y* + 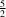, −*z* + 1. Displacement ellipsoids are drawn at the 50% probability level. [Further symmetry codes are: (ii) −*x*, −*y* + 2, −*z* + 1; (iii) *x*, *y* + 1, *z*; (iv) *x* + 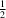, *y* + 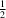, *z*; (v) −*x* + 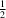, −*y* + 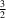, −*z* + 1; (vi) *x* + 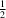, *y* + 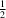, *z*.]

Reaction of **H_4_L1** with Zn(NO_3_)_2_ in the presence of a 1 *M* potassium acetate buffer led to the formation of colourless crystals that proved to be a dipotassium–organic framework (**K_2_H_2_L1**); see Fig. 8 ▸. The asymmetric unit consists of half a di-deprotonated ligand mol­ecule located about an inversion center, and two half potassium ions located on inversion centers. The K^+^ ions are linked to the O atoms of the acid groups and both K^+^ ions have a coordination number of six (KO_6_) and have edge-sharing bipyramidal geometries. The K^+^ ions are bridged by atoms O1 and O3, forming chains propagating along the *b*-axis direction (Fig. 7 ▸ *b*). The K⋯O bond lengths vary between 2.6682 (12) and 2.8099 (14) Å (Table 3 ▸). Here, the difference between the K⋯O(C=O) and K⋯O(O^−^) bond lengths is much less significant (Table 3 ▸).

The K⋯O bond lengths in the **KH_3_L1** and **K_2_H_2_L1** frameworks are close to those observed for similar compounds; see §*6 Database survey*. The conformation of one of the –CH_2_—S—CH_2_—CH_2_– side chains (involving atom S1) of the organic anion are similar, and similar to that in **H_4_L1_B** (Fig. 5 ▸ *b*), while the conformation of the second (involving atom S2) differs significantly (Fig. 5 ▸ *c* and *d*, and Table 1 ▸).

## Supra­molecular features   

**Figure 9:**
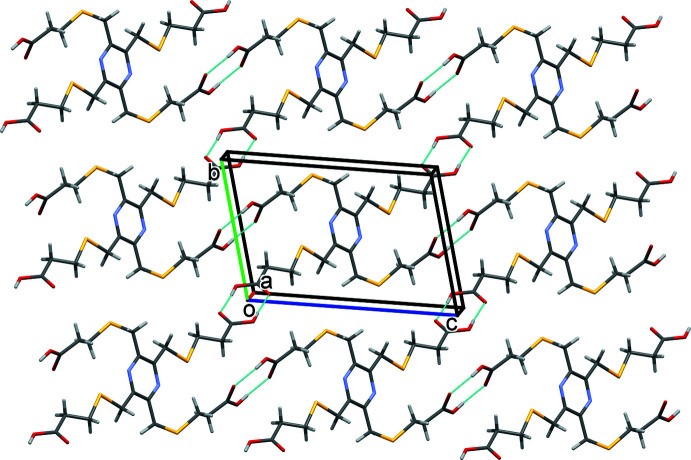
A view along the *a* axis of the crystal packing of **H_4_L1_A**. The hydrogen bonds are shown as dashed lines (see Table 4 ▸).

In the crystal of **H_4_L1_A**, mol­ecules are linked by pairs of O—H⋯O hydrogen bonds, forming classical carb­oxy­lic acid inversion dimers enclosing 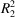
(8) loops (Fig. 9 ▸ and Table 4 ▸). These inter­actions lead to the formation of layers lying parallel to the *bc* plane. The layers are linked by C—H⋯O hydrogen bonds (Table 4 ▸), forming a supra­molecular framework.

**Figure 10:**
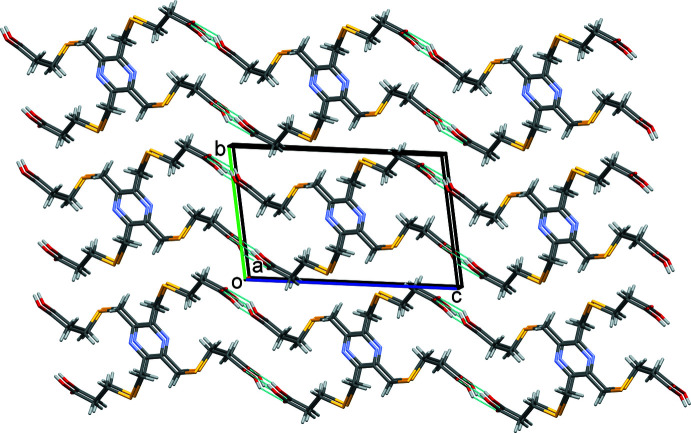
A view along the *a*-axis of the crystal packing of **H_4_L1_B**. Only atoms of the major component are shown. The hydrogen bonds are shown as dashed lines (see Table 5 ▸).

In the crystal of **H_4_L1_B**, mol­ecules are linked by pairs of O—H⋯O hydrogen bonds, forming chains propagating along the *c*-axis direction and enclosing 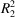
(8) loops (Fig. 10 ▸ and Table 5 ▸). There are no other significant directional contacts present in the crystal.

**Figure 11:**
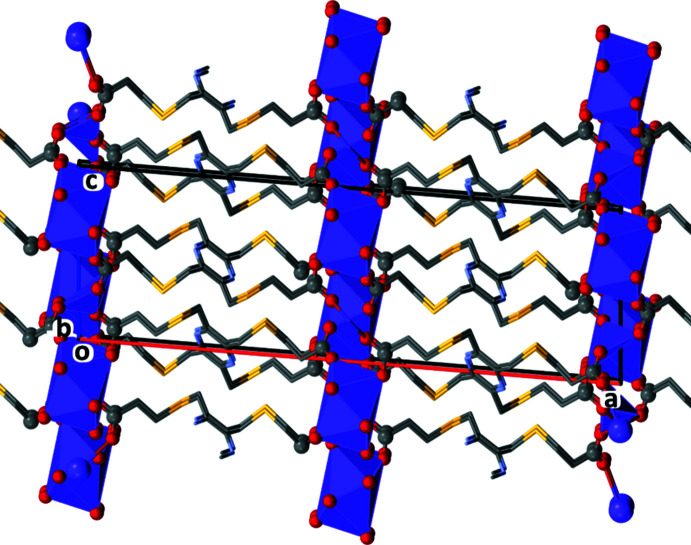
A view along the *b* axis of the crystal packing of complex **KH_3_L1**. For clarity, the H atoms have been omitted.

In both **KH_3_L1** and **K_2_H_2_L1**, the organic anions are arranged as rungs of parallel ladders, so forming the framework structures, as shown in Figs. 11 ▸ and 12 ▸, respectively. The frameworks are reinforced by O—H⋯O, C—H⋯O and C—H⋯N hydrogen bonds (Tables 6 ▸ and 7 ▸, respectively).

## Hirshfeld surface analysis and two-dimensional fingerprint plots for **H_4_L1_A**, and **H_4_L1_B**   

The Hirshfeld surface analysis (Spackman & Jayatilaka, 2009 ▸) and the associated two-dimensional fingerprint plots (McKinnon *et al.*, 2007 ▸) were performed with *CrystalExplorer17* (Turner *et al.*, 2017 ▸) following the protocol of Tiekink and collaborators (Tan *et al.*, 2019 ▸).

**Figure 13:**
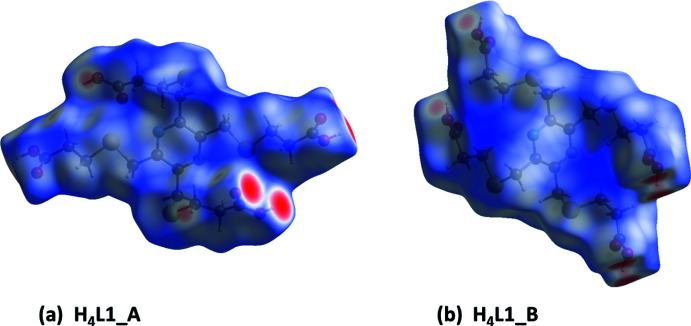
The Hirshfeld surfaces of compounds (*a*) **H_4_L1_A** and (*b*) **H_4_L1_B**, mapped over *d*_norm_ in the colour ranges of −0.7146 to 1.2167 and −0.6847 to 1.3548 au., respectively.

The Hirshfeld surfaces are colour-mapped with the normalized contact distance, *d*_norm_, varying from red (distances shorter than the sum of the van der Waals radii) through white to blue (distances longer than the sum of the van der Waals radii). The Hirshfeld surfaces (HS) of **H_4_L1_A**, and **H_4_L1_B** mapped over *d*_norm_ are given in Fig. 13 ▸. The most significant short contacts in the crystal structures of the two polymorphs are given in Table 8 ▸. The large red spots in Fig. 13 ▸ *a* and *b* concern the O—H⋯O hydrogen bonds in the crystal structures of both compounds.

**Figure 14:**
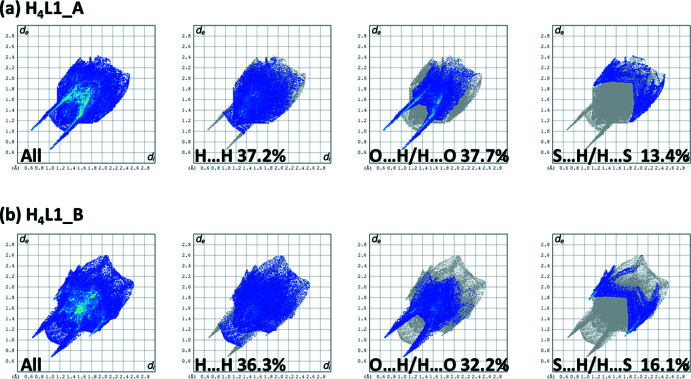
The full two-dimensional fingerprint plots for compounds (*a*) **H_4_L1_A** and (*b*) **H_4_L1_B**, and those delineated into H⋯H, O⋯H/H⋯O and S⋯H/H⋯S contacts.

The percentage contributions of inter-atomic contacts to the HS for both compounds are compared in Table 9 ▸. The two-dimensional fingerprint plots for compounds **H_4_L1_A**, and **H_4_L1_B** are shown in Fig. 14 ▸. They reveal that the principal contributions to the overall HS involve H⋯H contacts at 37.2 and 36.3%, respectively, and O⋯H/H⋯O contacts at, respectively, 37.7 and 32.2%.

The third most important contribution to the HS is from the S⋯H/H⋯S contacts at 13.4 and 16.1%, for **H_4_L1_A**, and **H_4_L1_B**, respectively. These are followed by C⋯H/H⋯H contacts at, respectively, 4.5 and 4.9%. The N⋯H/H⋯N contacts contribute, respectively, 3.0 and 2.5%.

## Energies frameworks for **H_4_L1_A**, and **H_4_L1_B**   

**Figure 15:**
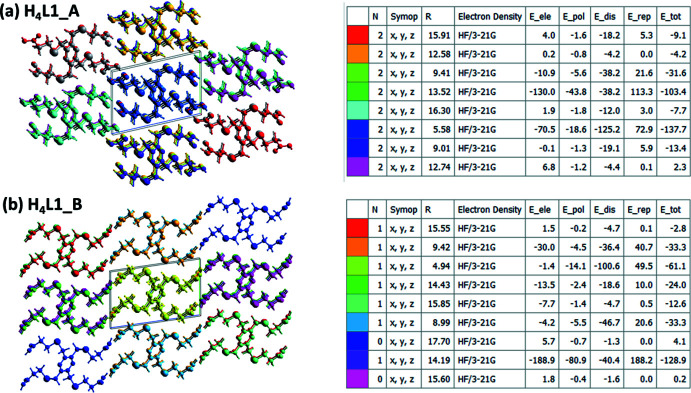
The colour-coded inter­action mappings within a radius of 6 Å of a central reference mol­ecule for (*a*) **H_4_L1_A** and (*b*) **H_4_L1_B**.

The colour-coded inter­action mappings within a radius of 6 Å of a central reference mol­ecule for **H_4_L1_A**, and **H_4_L1_B**, are given in Fig. 15 ▸. Full details of the various contributions to the total energy (*E*_tot_) are also included there; see Tan *et al.* (2019 ▸) for an explanation of the various parameters.

**Figure 16:**
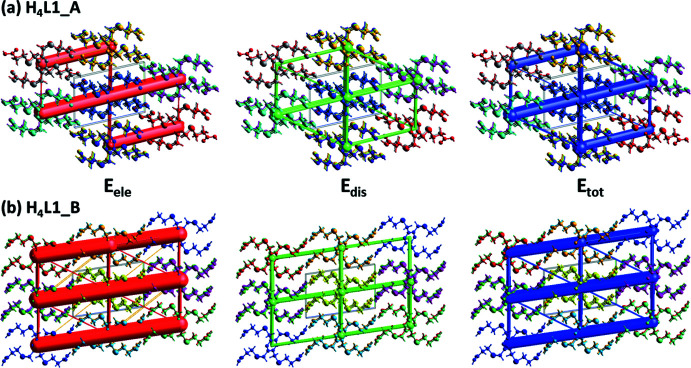
The energy frameworks calculated for (*a*) **H_4_L1_A** and (*b*) **H_4_L1_B**, both viewed along the *b-*axis direction, showing the electrostatic potential forces (*E*_ele_), the dispersion forces (*E*_dis_) and the total energy diagrams (*E*_tot_).

A comparison of the energy frameworks calculated for **H_4_L1_A**, and **H_4_L1_B**, showing the electrostatic potential forces (*E*_ele_), the dispersion forces (*E*_dis_) and the total energy diagrams (E_tot_), are shown in Fig. 16 ▸. The energies were obtained by using the wave function at the HF/3-21G level of theory. The cylindrical radii are proportional to the relative strength of the corresponding energies (Turner *et al.*, 2017 ▸; Tan *et al.*, 2019 ▸). They have been adjusted to the same scale factor of 80 with a cut-off value of 5 kJ mol^−1^ within a radius of 6 Å of a central reference mol­ecule. It can be seen that for both polymorphs the major contribution to the inter­molecular inter­actions is from electrostatic potential forces (*E*_ele_), reflecting the presence of the classical O—H⋯O hydrogen bonds.

## Database survey   

A search of the Cambridge Structural Database (CSD, Version 5.42, last update February 2021; Groom *et al.*, 2016 ▸) for tetrakis-substituted pyrazine carb­oxy­lic acids gave results for only two such ligands, *viz*. 2,3,5,6-pyrazine­tetra­carb­oxy­lic acid (**pztca**) and 2,3,5,6-tetra­kis­(4-carb­oxy­phen­yl)pyrazine (**pztba**). Ligand **pztba** has been shown to be extremely successful in forming metal–organic frameworks (Jiang *et al.*, 2017 ▸; Wang *et al.*, 2019 ▸).

**Figure 17:**
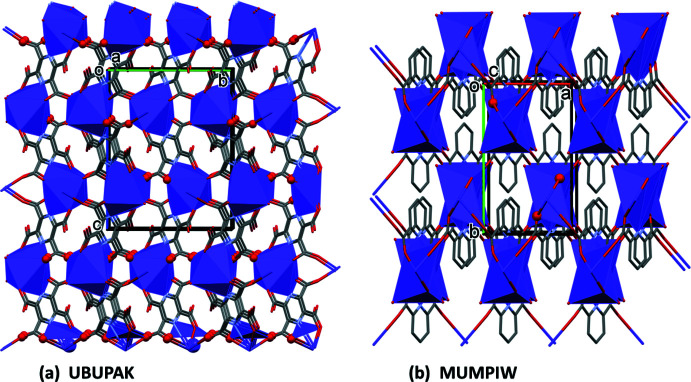
(*a*) A view along the *a* axis of the potassium–organic framework of UBUPAK (Masci *et al.*, 2010 ▸) and (*b*) a view along the *c* axis of the potassium–organic framework of MUMPIW (Li *et al.*, 2020 ▸).

Potassium salts of carb­oxy­lic acids are relatively common. A search for potassium salts of purely organic carb­oxy­lic acids and excluding hydrates, yielded over 200 hits. The potassium salt of **pztca** has been reported, *viz. catena*-[(μ_4_-3,5,6-tri­carb­oxy­pyrazine-2-carboxyl­ato)potassium] (CSD refcode UBUPAK; Masci *et al.*, 2010 ▸). The structure of UBUPAK is that of a potassium–organic framework (Fig. 17 ▸ *a*). The asymmetric unit consists of half a mono-deprotonated ligand mol­ecule located about an inversion center, and half a potassium ion. The carb­oxy H atom is disordered by symmetry, similar to the situation in the structure of **KH_3_L1**. Here the K⋯O bond lengths vary from 2.7951 (11) to 2.8668 (13) Å. The K^+^ cation has a coordination number of 8 (KO_8_) and a distorted dodeca­hedral geometry as in **KH_3_L1** (Fig. 7 ▸ *a* and 11).

The structure of the potassium salt of pyrazine-2,3-di­carb­oxy­lic acid (**pzdca**; Fig. 1 ▸), catena-[(μ_2_-3-carb­oxy­pyrazine-2-carboxyl­ato)-(μ_2_-pyrazine-2,3-di­carb­oxy­lic acid)di­aqua­potassium], has been reported (RISYIC; Tombul *et al.*, 2008 ▸). It has a polymer chain structure with the chains linked by O—H⋯O hydrogen bonds, forming a supra­molecular framework. Here the K⋯O bond lengths vary from 2.8772 (14) to 3.0898 (14) Å.

The structures of two potassium salts of 2,6-pyridine-di­carb­oxy­lic acid (**pydca**; Fig. 1 ▸) have been reported. They include, bis­(μ_2_-pyridine-2,6-di­carb­oxy­lic acid-*N,O,O′:O′*)-hexa­aqua­bis­(6-carb­oxy­pyridine-2-carboxyl­ato-*O*)dipotassium (HAMBEE; Santra *et al.*, 2011 ▸; HAMBEE01; Hayati *et al.*, 2017 ▸), and *catena*-[(μ-6-carb­oxy­pyridine-2-carboxyl­ato)potassium] (MUMPIW; Li *et al.*, 2020 ▸). HAMBEE is a binuclear complex, which is linked by O—H⋯O hydrogen bonds to form supra­molecular chains. The K⋯O bond lengths vary from 2.721 (2) to 3.054 (3) Å.

The structure of MUMPIW is that of a potassium-organic framework (Fig. 17 ▸ *b*), with the K⋯O bonds lengths varying from 2.8197 (14) to 3.0449 (15) Å. The K^+^ ion has a coordination number of seven (KO_6_N) and has an edge-sharing penta­gonal anti­prism geometry, forming chains (Fig. 17 ▸ *b*). This structure can be compared to that of **K_2_H_2_L1** where the two independent K^+^ ions, each with a coordination number of six (KO_6_), have edge-sharing bipyramidal geometries, also forming chains (Fig. 7 ▸ *b* and 12).

## Synthesis and crystallization   

The synthesis and crystal structure of the reagent tetra-2,3,5,6-bromo­methyl-pyrazine (TBr) have been reported (Ferigo *et al.*, 1994 ▸; Assoumatine & Stoeckli-Evans, 2014 ▸ [CSD refcode: TOJXUN]).

**Synthesis of 3,3′,3′′,3′′′-{[pyrazine-2,3,5,6-tetra­yltetra­kis(methyl­ene)]tetra­kis­(sulfanedi­yl)}tetra­propionic acid (H_4_L1):**

Mercaptopropionic acid (1.8795 g, 1.77 mol, 4 eq) was dissolved in 50 ml THF. A minimum amount of water (a few ml) was added to dissolve 1.4166 g (3.54 mol, 8 eq) of NaOH. The volume of the mixture was increased to 100 ml by adding THF and the reaction was stirred under reflux for 1 h. Then TBr (2 g, 4.42 mol, 1 eq) dissolved in 50 ml THF was added dropwise using an addition funnel. The mixture was stirred under reflux for 6 h. After drying under vacuum, the residue was dissolved in 50 ml of deionized water, and HCl puriss. was added dropwise until a clearly acid pH was obtained. This mixture was stirred at room temperature for 1–2 h. The yellow precipitate that formed was filtered off and washed with a minimum amount of water and then CHCl_3_. It was then dried under vacuum conditions. Recrystallization carried out with methanol gave pale-yellow crystals of **H_4_L1** (yield 88%, m.p. 466 K) that X-ray diffraction analysis indicated to be triclinic polymorph **H_4_L1_A**.

The presence of terephthalic acid in an equimolar qu­antity with **H_4_L1** in methanol gave colourless crystals of rather poor quality. However, X-ray diffraction analysis indicated that a second triclinic (*P* 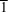) polymorph, **H_4_L1_B**, had been obtained.

**Spectroscopic and elemental analyses:**

*R*_f_: 0.77 (solvent: CH_3_OH).

^1^H NMR (CD_3_OD, 400 MHz), δ(ppm): 4.03 (*s*, 8H, H2), 2.78 (*t*, 8H, ^3^ *J*_(3,4)_ = 7.0, H3), 2.62 (*t*, 8H, ^3^ *J*_(4,3)_ = 7.0, H4).

^13^C NMR (CD_3_OD, 50 MHz), δ(ppm): 174.54 (4C, C5), 150.12 (4C, C1), 34.29 (4C, C4), 33.64 (4C, C2), 26.65 (4C, C3).

Elemental Analysis for C_20_H_28_N_2_O_8_S_4_, *M*_w_ = 552.71 g mol^−1^: Calculated: C 43.46, H 5.11, N 5.07%. Found: C 43.40, H 5.17, N 4.87%.

ESI–MS, *m*/*z*: 591.04 [*M* + K]^+^; 575.06 [*M* + Na]^+^; 553.08 [*M* + H]^+^; 471.07; 449.09.

IR (KBr disc, cm^−1^) ν: 2926(*s*), 2666(*m*), 2590(*s*), 1693(*s*), 1429(*s*), 1406(*s*), 1340(*m*), 1270(s), 1200(*s*), 1163(*m*), 1134(*s*), 1107(*m*), 1055(*w*), 918(*s*), 658(*m*), 489(*m*).

**Synthesis of poly[(μ-3-{[(3,5,6-tris­{[(2-carb­oxy­eth­yl)sulfan­yl]meth­yl}pyrazin-2-yl)meth­yl]sulfan­yl}propano­ate)potas­sium] (KH_3_L1):**

Hg(NO_3_)_2_ (45.0 mg, 0.109 mmol, 2 eq) and **H_4_L1** (30 mg, 0.054 mmol, 1 eq) were mixed together in 20 ml of a 1 *M* potassium acetate buffer. The mixture was left at 323 K under stirring and nitro­gen conditions for 1 h. The mixture was then filtered and left to evaporate in air for six weeks. Colourless plate-like crystals were obtained, which were shown to be a potassium–organic framework.

IR (KBr disc, cm^−1^) ν: 3422(*m*), 2922(*m*), 1713(*m*), 1580(*s*), 1399(*s*), 1247(*m*), 1190(*m*), 1152(*m*), 1114(*m*), 811(*m*), 787(*m*).

**Synthesis of poly[(μ-3,3′-{[(3,6-bis­{[(2-carb­oxy­eth­yl)sulf­an­yl]meth­yl}pyrazine-2,5-di­yl)bis­(methyl­ene)]bis­(sulfanedi­yl)}dipropionato)dipotassium] (K_2_H_2_L1):**

Zn(NO_3_)_2_ (28.4 mg, 0.109 mmol, 2 eq) and **H_4_L1** (30 mg, 0.054 mmol, 1eq) were mixed together in 20 ml of a 1M potassium acetate buffer. The mixture was left at 323 K under stirring and nitro­gen for 1 h. The mixture was then filtered and left to evaporate in air for 6 weeks. Colourless plate-like crystals were obtained, which proved to be a dipotassium-organic framework.

IR (KBr disc, cm^−1^) ν: 3401(*m*), 1579(*s*), 1401(*s*), 1303(*m*).

## Refinement   

Crystal data, data collection and structure refinement details are summarized in Table 10 ▸.

For **H_4_L1_A**, **KH_3_L1** and **K_2_H_2_L1**, the various –CO_2_H H atoms were located in difference-Fourier maps and freely refined. For **H_4_L1_B**, the –CO_2_H H atoms were difficult to locate, probably due to the poor quality of the crystal and the disorder in the side chain (atoms C8/C8*B*, C9/C9*B*, C10/C10*B*, O3/O3*B*, O4/O4*B*; Fig. 4 ▸ *b*). They were therefore included in calculated positions assuming the formation of carb­oxy­lic acid dimers; O—H = 0.82 Å and refined as riding with *U*_iso_(H) = 1.5*U*_eq_(O).

As in the K^+^ salt of pyrazine tetra­carb­oxy­lic acid (UBUPAK; Masci *et al.*, 2010 ▸), the carb­oxy H atom in **KH_3_L1** is disordered by symmetry, hence the H atom on O3 was given an occupancy factor of 0.5 to balance the charges.

For all four compounds, the C-bound H atoms were included in calculated positions and treated as riding on their parent C atom with C—H = 0.97 Å and *U*_iso_(H) = 1.2*U*_eq_(C).

For **H_4_L1_A** and **H_4_L1_B**, the alert _diffrn_reflns_point_group_measured_fraction_full value (0.94 and 0.93, respectively) below minimum (0.95) was given. For **H_4_L1_A** it involves 131 random reflections out of a total of 2180, *viz.* 6.0%, while for **H_4_L1_B** it involves 158 random reflections out of a total of 2184, *viz.* 7.2%.

For **H_4_L1_A**, **H_4_L1_B** and **K_2_H_2_L1** the multiplicity of reflections was 2 or less and so an empirical absorption correction was applied.

## Supplementary Material

Crystal structure: contains datablock(s) H4L1A, H4L1B, KH3L1, K2H2L1, Global. DOI: 10.1107/S2056989021003479/pk2656sup1.cif

Structure factors: contains datablock(s) H4L1A. DOI: 10.1107/S2056989021003479/pk2656H4L1Asup2.hkl

Structure factors: contains datablock(s) H4L1B. DOI: 10.1107/S2056989021003479/pk2656H4L1Bsup3.hkl

Structure factors: contains datablock(s) KH3L1. DOI: 10.1107/S2056989021003479/pk2656KH3L1sup4.hkl

Structure factors: contains datablock(s) K2H2L1. DOI: 10.1107/S2056989021003479/pk2656K2H2L1sup5.hkl

Supporting information file. DOI: 10.1107/S2056989021003479/pk2656H4L1Asup6.cml

Supporting information file. DOI: 10.1107/S2056989021003479/pk2656H4L1Bsup7.cml

Supporting information file. DOI: 10.1107/S2056989021003479/pk2656KH3L1sup8.cml

Supporting information file. DOI: 10.1107/S2056989021003479/pk2656K2H2L1sup9.cml

CCDC references: 2074770, 2074769, 2074768, 2074767

Additional supporting information:  crystallographic information; 3D view; checkCIF report

## Figures and Tables

**Figure 12:**
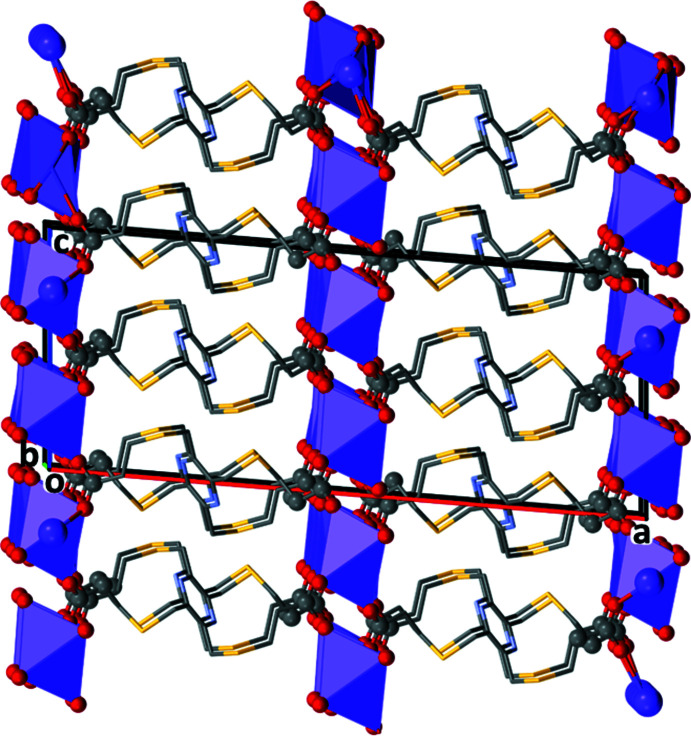
A view along the *b* axis of the crystal packing of complex **K_2_H_2_L1**. For clarity, the H atoms have been omitted.

**Table 1:** Selected torsion angles (°) along the C_ar_—CH_2_—S—CH_2_—CH_2_—CO_2_H side chains in compounds **H_4_L1_A**, **H_4_L1_B**, **KH_3_L1** and **K_2_H_2_L1**

Torsion angle	**H_4_L1_A**	**H_4_L1_B**	**KH_3_L1**	**K_2_H_2_L1**
C1—C3—S1—C4	174.1 (2)	−72.6 (4)	−72.32)	−65.81 (15)
C3—S1—C4—C5	−155.3 (2)	−86.7 (4)	−90.3 (2)	−87.72 (15)
S1—C4—C5—C6	−167.9 (2)	−65.0 (6)	−76.4 (3)	−73.19 (18)
C2—C7—S2—C8	57.6 (2)	−66.8 (4)	−62.3 (2)	−67.34 (15)
C7—S2—C8—C9	65.7 (2)	−178.1 (5)	−77.5 (2)	97.89 (15)
S2—C8—C9—C10	174.8 (2)	−172.5 (5)	−173.8 (2)	174.51 (12)

**Table 2:** Selected bond lengths (Å) for **KH_3_L1** [Chem scheme1]

K1—O1	2.828 (2)	K1—O3^ii^	2.682 (2)
K1—O2^i^	3.056 (3)	K1—O4^iii^	3.069 (3)

Symmetry codes: (i) x, -y, z-{\script{1\over 2}}; (ii) -x+{\script{1\over 2}}, y-{\script{1\over 2}}, -z-{\script{1\over 2}}; (iii) x+{\script{1\over 2}}, -y-{\script{1\over 2}}, z-{\script{1\over 2}}.

**Table 3:** Selected bond lengths (Å) for **K_2_H_2_L1** [Chem scheme1]

K1—O1^i^	2.7084 (14)	K2—O1	2.7132 (13)
K1—O2	2.6682 (12)	K2—O3^iii^	2.6682 (13)
K1—O3^ii^	2.8099 (14)	K2—O4^ii^	2.7209 (12)

Symmetry codes: (i) x, -y+2, z+{\script{1\over 2}}; (ii) x-{\script{1\over 2}}, y-{\script{1\over 2}}, z; (iii) -x+{\script{1\over 2}}, -y+{\script{3\over 2}}, -z+1.

**Table 4:** Hydrogen-bond geometry (Å, °) for **H_4_L1_A** [Chem scheme1]

*D*—H⋯*A*	*D*—H	H⋯*A*	*D*⋯*A*	*D*—H⋯*A*
O2—H2*O*⋯O1^i^	0.87 (2)	1.80 (2)	2.667 (3)	172 (5)
O4—H4*O*⋯O3^ii^	0.83 (2)	1.85 (2)	2.673 (3)	175 (5)
C5—H5*A*⋯O3^iii^	0.97	2.55	3.405 (4)	147
C8—H8*A*⋯O4^iv^	0.97	2.40	3.308 (4)	156

Symmetry codes: (i) -x-1, -y, -z; (ii) -x+1, -y+1, -z; (iii) x-1, y, z; (iv) x+1, y, z.

**Table 5:** Hydrogen-bond geometry (Å, °) for **H_4_L1_B** [Chem scheme1]

*D*—H⋯*A*	*D*—H	H⋯*A*	*D*⋯*A*	*D*—H⋯*A*
O2—H2*O*⋯O3^i^	0.82	1.94	2.66 (1)	146
O2—H2*O*⋯O3*B* ^i^	0.82	2.20	2.77 (3)	127
O4—H4*O*⋯O1^ii^	0.82	1.88	2.66 (1)	158
O4*B*—H4*OB*⋯O1^ii^	0.82	1.86	2.67 (4)	170

Symmetry codes: (i) x, y, z+1; (ii) x, y, z-1.

**Table 6:** Hydrogen-bond geometry (Å, °) for **KH_3_L1** [Chem scheme1]

*D*—H⋯*A*	*D*—H	H⋯*A*	*D*⋯*A*	*D*—H⋯*A*
O4—H4*O*⋯O1^iv^	0.80 (5)	1.86 (5)	2.661 (3)	180 (7)
O2—H20⋯O2^v^	1.24 (1)	1.24 (1)	2.436 (3)	159 (7)
C4—H4*A*⋯N1	0.99	2.52	3.340 (4)	140
C4—H4*B*⋯O3^vi^	0.99	2.49	3.114 (4)	121
C5—H5*B*⋯O2^i^	0.99	2.60	3.467 (4)	146
C7—H7*B*⋯N1^vii^	0.99	2.60	3.454 (4)	144
C9—H9*A*⋯O3^vii^	0.99	2.58	3.465 (4)	149

Symmetry codes: (i) x, -y, z-{\script{1\over 2}}; (iv) x-{\script{1\over 2}}, y+{\script{1\over 2}}, z; (v) -x+1, y, -z+{\script{1\over 2}}; (vi) -x+{\script{1\over 2}}, -y+{\script{1\over 2}}, -z; (vii) x, -y, z+{\script{1\over 2}}.

**Table 7:** Hydrogen-bond geometry (Å, °) for **K_2_H_2_L1** [Chem scheme1]

*D*—H⋯*A*	*D*—H	H⋯*A*	*D*⋯*A*	*D*—H⋯*A*
O4—H4*O*⋯O2^iv^	0.85 (2)	1.61 (2)	2.4637 (16)	177 (3)
C4—H4*A*⋯N1	0.99	2.44	3.266 (2)	141
C8—H8*A*⋯O3^v^	0.99	2.53	3.436 (2)	151

Symmetry codes: (iv) x+{\script{1\over 2}}, y+{\script{1\over 2}}, z; (v) x, -y+2, z-{\script{1\over 2}}.

**Table 8:** Short contacts (Å) in the crystal structures of **H_4_L1_A** and **H_4_L1_B** *^*a*^*

Atom 1	Atom 2	Length	Length − VdW	Symm. op. 1	Symm. op. 2
**H_4_L1_A**					
O1	H2*O*	1.798	−0.922	*x*, *y*, *z*	−1 − *x*, −*y*, −*z*
O3	H4*O*	1.843	−0.877	*x*, *y*, *z*	1 − *x*, 1 − *y*, −*z*
O1	O2	2.667	−0.373	*x*, *y*, *z*	−1 − *x*, −*y*, −*z*
O3	O4	2.673	−0.367	*x*, *y*, *z*	1 − *x*, 1 − *y*, −*z*
O4	H8*A*	2.399	−0.321	*x*, *y*, *z*	−1 + *x*, *y*, *z*
O2	O4	3.015	−0.025	*x*, *y*, *z*	−*x*, 1 − *y*, −*z*
C6	H2*O*	2.667	−0.233	*x*, *y*, *z*	−1 − *x*, −*y*, −*z*
C10	H4*O*	2.668	−0.232	*x*, *y*, *z*	1 − *x*, 1 − *y*, −*z*
H5*A*	O3	2.549	−0.171	*x*, *y*, *z*	−1 + *x*, *y*, *z*
H4*O*	H4*O*	2.371	−0.029	*x*, *y*, *z*	1 − *x*, 1 − *y*, −*z*
H2*O*	H2*O*	2.389	−0.011	*x*, *y*, *z*	−1 − *x*, −*y*, −*z*
N1	H3*A*	2.807	0.057	*x*, *y*, *z*	1 − *x*, 1 − *y*, 1 − *z*
O4	C8	3.308	0.088	*x*, *y*, *z*	−1 + *x*, *y*, *z*
O2	H8*A*	2.820	0.100	*x*, *y*, *z*	1 − *x*, 1 − *y*, −*z*
					
**H_4_L1_B*^*a*^***					
H4*O*	O1	1.879	−0.841	*x*, *y*, *z*	*x*, *y*, −1 + *z*
O4	O1	2.658	−0.382	*x*, *y*, *z*	*x*, *y*, −1 + *z*
O3	O2	2.663	−0.377	*x*, *y*, *z*	*x*, *y*, −1 + *z*
H4*O*	C6	2.580	−0.320	*x*, *y*, *z*	*x*, *y*, −1 + *z*
O4	O2	2.799	−0.241	*x*, *y*, *z*	−1 + *x*, *y*, −1 + *z*
H4*O*	H2*O*	2.173	−0.227	*x*, *y*, *z*	*x*, *y*, −1 + *z*
O1	O2	2.982	−0.058	*x*, *y*, *z*	−1 + *x*, *y*, *z*
S1	H3*A*	2.951	−0.049	*x*, *y*, *z*	−1 + *x*, *y*, *z*
S1	S2	3.590	−0.010	*x*, *y*, *z*	1 − *x*, −*y*, 1 − *z*
O4	O3	3.041	0.001	*x*, *y*, *z*	−1 + *x*, *y*, *z*
S2	S2	3.613	0.013	*x*, *y*, *z*	1 − *x*, −*y*, 1 − *z*
H8*A*	O3	2.749	0.029	*x*, *y*, *z*	−1 + *x*, *y*, *z*
S1	H5*A*	3.047	0.047	*x*, *y*, *z*	−1 + *x*, *y*, *z*
H4*O*	O2	2.775	0.055	*x*, *y*, *z*	−1 + *x*, *y*, −1 + *z*
O4	H2*O*	2.776	0.056	*x*, *y*, *z*	−1 + *x*, *y*, −1 + *z*
C10	H2*O*	2.960	0.060	*x*, *y*, *z*	2 − *x*, −*y*, 1 − *z*
O3	H2*O*	2.796	0.076	*x*, *y*, *z*	2 − *x*, −*y*, 1 − *z*
H7*B*	C3	2.974	0.074	*x*, *y*, *z*	−1 + *x*, *y*, *z*
S2	H7*B*	3.082	0.082	*x*, *y*, *z*	1 − *x*, −*y*, 1 − *z*
O2	H5*B*	2.802	0.082	2 − *x*, 1 − *y*, 1 − *z*	−1 + *x*, *y*, −1 + *z*
S1	H9*A*	3.085	0.085	*x*, *y*, *z*	1 − *x*, −*y*, 1 − *z*

Note: (*a*) major component of **H_4_L1_B**.

**Table 9:** Percentage contributions of inter-atomic contacts to the Hirshfeld surfaces of **H_4_L1_A** and **H_4_L1_B*^*a*^***

Contact	% contribution	% contribution
	**H_4_L1_A**	**H_4_L1_B** *^*a*^*
H⋯H	37.2	36.3
O⋯H/H⋯O	37.7	32.2
S⋯H/H⋯S	13.4	16.1
C⋯H/H⋯C	4.5	4.9
N⋯H/H⋯N	3.0	2.5
C⋯N	0	0.8
C⋯O	1.0	0.7
C⋯S	1.2	0
N⋯S	0.4	0.4
O⋯O	1.3	4.9
O⋯S	0.2	0
S⋯S	0.2	1.2

Note: (*a*) major component of **H_4_L1_B**.

**Table 10:** Experimental details

	**H_4_L1_A**	**H_4_L1_B**	**KH_3_L1**	**K_2_H_2_L1**
Crystal data				
Chemical formula	C_20_H_28_N_2_O_8_S_4_	C_20_H_28_N_2_O_8_S_4_	[K(C_20_H_27_N_2_O_8_S_4_)]	[K_2_(C_20_H_26_N_2_O_8_S_4_)]
*M*_r_	552.68	552.68	590.77	628.87
Crystal system, space group	Triclinic, *P*\overline{1}	Triclinic, *P*\overline{1}	Monoclinic, *C*2/*c*	Monoclinic, *C*2/*c*
Temperature (K)	293	293	153	153
*a*, *b*, *c* (Å)	5.5843 (8), 9.0061 (14), 12.739 (2)	4.9424 (17), 8.993 (3), 14.190 (6)	30.080 (4), 8.4716 (10), 9.5908 (12)	27.908 (2), 8.2916 (6), 11.3035 (9)
α, β, γ (°)	101.537 (18), 94.313 (18), 103.701 (17)	96.96 (3), 97.14 (3), 100.72 (3)	90, 94.717 (11), 90	90, 94.753 (6), 90
*V* (Å^3^)	604.80 (17)	608.1 (4)	2435.7 (6)	2606.7 (3)
*Z*	1	1	4	4
Radiation type	Mo *K*α	Mo *K*α	Mo *K*α	Mo *K*α
μ (mm^−1^)	0.44	0.44	0.61	0.73
Crystal size (mm)	0.35 × 0.30 × 0.05	0.50 × 0.50 × 0.05	0.50 × 0.50 × 0.10	0.50 × 0.50 × 0.05

Data collection				
Diffractometer	Stoe IPDS 1	Stoe IPDS 2	Stoe IPDS 2	Stoe IPDS 2
Absorption correction	Empirical (using intensity measurements) (*ShxAbs*; Spek, 2020 ▸)	Empirical (using intensity measurements) (*ShxAbs*; Spek, 2020 ▸)	Multi-scan (*MULABS*; Spek, 2020 ▸)	Empirical (using intensity measurements) (*ShxAbs*; Spek, 2020 ▸)
*T*_min_, *T*_max_	0.647, 0.897	0.144, 0.616	0.640, 1.000	0.416, 0.803
No. of measured, independent and observed [*I* > 2σ(*I*)] reflections	4709, 2194, 1452	4152, 2201, 1537	10309, 2084, 1646	19423, 3646, 3175
*R*_int_	0.058	0.080	0.064	0.042
(sin θ/λ)_max_ (Å^−1^)	0.615	0.617	0.590	0.695

Refinement				
*R*[*F* ^2^ > 2σ(*F* ^2^)], *wR*(*F* ^2^), *S*	0.041, 0.097, 0.88	0.071, 0.208, 1.05	0.039, 0.106, 1.02	0.037, 0.103, 1.05
No. of reflections	2194	2201	2084	3646
No. of parameters	162	173	165	167
No. of restraints	2	6	0	1
H-atom treatment	H atoms treated by a mixture of independent and constrained refinement	H-atom parameters constrained	H atoms treated by a mixture of independent and constrained refinement	H atoms treated by a mixture of independent and constrained refinement
Δρ_max_, Δρ_min_ (e Å^−3^)	0.35, −0.28	0.47, −0.39	0.26, −0.36	0.76, −0.51

Computer programs: *EXPOSE*, *CELL* and *INTEGRATE* in *IPDS*-I (Stoe & Cie, 2000 ▸), *X-AREA* and *X-RED32* (Stoe & Cie, 2002 ▸), *SHELXS97* (Sheldrick, 2008 ▸), *SHELXL2018*/3 (Sheldrick, 2015 ▸), *PLATON* (Spek, 2020 ▸) *Mercury* (Macrae *et al.*, 2020 ▸) and *publCIF* (Westrip, 2010 ▸).

## supplementary crystallographic information

### 3,3',3'',3'''-{[Pyrazine-2,3,5,6-tetrayltetrakis(methylene))tetrakis(sulfanediyl]}tetrapropionic acid (H4L1A) Crystal data

**Table:**

C_20_H_28_N_2_O_8_S_4_	*Z* = 1
*M**_r_* = 552.68	*F*(000) = 290
Triclinic, *P*1	*D*_x_ = 1.517 Mg m^−^^3^
*a* = 5.5843 (8) Å	Mo *K*α radiation, λ = 0.71073 Å
*b* = 9.0061 (14) Å	Cell parameters from 3225 reflections
*c* = 12.739 (2) Å	θ = 2.4–25.9°
α = 101.537 (18)°	µ = 0.44 mm^−^^1^
β = 94.313 (18)°	*T* = 293 K
γ = 103.701 (17)°	Plate, pale-yellow
*V* = 604.80 (17) Å^3^	0.35 × 0.30 × 0.05 mm

### 3,3',3'',3'''-{[Pyrazine-2,3,5,6-tetrayltetrakis(methylene))tetrakis(sulfanediyl]}tetrapropionic acid (H4L1A) Data collection

**Table:**

STOE IPDS 1 diffractometer	2194 independent reflections
Radiation source: fine-focus sealed tube	1452 reflections with *I* > 2σ(*I*)
Plane graphite monochromator	*R*_int_ = 0.058
φ rotation scans	θ_max_ = 25.9°, θ_min_ = 2.4°
Absorption correction: empirical (using intensity measurements) (*ShxAbs*; Spek, 2020)	*h* = −6→6
*T*_min_ = 0.647, *T*_max_ = 0.897	*k* = −11→11
4709 measured reflections	*l* = −14→15

### 3,3',3'',3'''-{[Pyrazine-2,3,5,6-tetrayltetrakis(methylene))tetrakis(sulfanediyl]}tetrapropionic acid (H4L1A) Refinement

**Table:**

Refinement on *F*^2^	Primary atom site location: structure-invariant direct methods
Least-squares matrix: full	Secondary atom site location: difference Fourier map
*R*[*F*^2^ > 2σ(*F*^2^)] = 0.041	Hydrogen site location: mixed
*wR*(*F*^2^) = 0.097	H atoms treated by a mixture of independent and constrained refinement
*S* = 0.88	*w* = 1/[σ^2^(*F*_o_^2^) + (0.0478*P*)^2^] where *P* = (*F*_o_^2^ + 2*F*_c_^2^)/3
2194 reflections	(Δ/σ)_max_ < 0.001
162 parameters	Δρ_max_ = 0.35 e Å^−^^3^
2 restraints	Δρ_min_ = −0.28 e Å^−^^3^

### 3,3',3'',3'''-{[Pyrazine-2,3,5,6-tetrayltetrakis(methylene))tetrakis(sulfanediyl]}tetrapropionic acid (H4L1A) Special details

**Table:**

Geometry. All esds (except the esd in the dihedral angle between two l.s. planes) are estimated using the full covariance matrix. The cell esds are taken into account individually in the estimation of esds in distances, angles and torsion angles; correlations between esds in cell parameters are only used when they are defined by crystal symmetry. An approximate (isotropic) treatment of cell esds is used for estimating esds involving l.s. planes.

### 3,3',3'',3'''-{[Pyrazine-2,3,5,6-tetrayltetrakis(methylene))tetrakis(sulfanediyl]}tetrapropionic acid (H4L1A) Fractional atomic coordinates and isotropic or equivalent isotropic displacement parameters (Å^2^)

**Table:**

	*x*	*y*	*z*	*U*_iso_*/*U*_eq_	
S1	0.40170 (15)	0.39121 (9)	0.29698 (6)	0.0312 (2)	
S2	1.27548 (15)	0.86290 (9)	0.37908 (6)	0.0288 (2)	
O1	−0.3381 (5)	0.0716 (3)	0.12207 (17)	0.0435 (6)	
O2	−0.2098 (5)	0.1064 (3)	−0.03567 (17)	0.0409 (6)	
H2O	−0.363 (5)	0.052 (5)	−0.059 (4)	0.098 (19)*	
O3	0.7761 (4)	0.5277 (3)	0.08084 (16)	0.0379 (6)	
O4	0.5045 (5)	0.6736 (3)	0.09232 (19)	0.0422 (6)	
H4O	0.422 (8)	0.607 (4)	0.039 (3)	0.087 (17)*	
N1	0.8353 (4)	0.5540 (3)	0.44074 (17)	0.0198 (5)	
C1	0.7859 (5)	0.4024 (3)	0.4451 (2)	0.0194 (6)	
C2	1.0447 (5)	0.6523 (3)	0.4952 (2)	0.0183 (6)	
C3	0.5460 (5)	0.2957 (3)	0.3850 (2)	0.0248 (6)	
H3A	0.434405	0.264980	0.436177	0.030*	
H3B	0.577409	0.201607	0.342827	0.030*	
C4	0.1473 (6)	0.2234 (4)	0.2308 (2)	0.0328 (7)	
H4A	0.202811	0.128008	0.222673	0.039*	
H4B	0.011542	0.214305	0.274306	0.039*	
C5	0.0600 (6)	0.2449 (4)	0.1219 (2)	0.0347 (8)	
H5A	0.041788	0.350865	0.129466	0.042*	
H5B	0.186183	0.233148	0.074799	0.042*	
C6	−0.1807 (6)	0.1321 (3)	0.0698 (2)	0.0291 (7)	
C7	1.0877 (6)	0.8196 (3)	0.4857 (2)	0.0239 (6)	
H7A	1.170302	0.887935	0.554088	0.029*	
H7B	0.928434	0.841937	0.471163	0.029*	
C8	1.0935 (6)	0.7220 (4)	0.2615 (2)	0.0277 (7)	
H8A	1.187763	0.725553	0.200717	0.033*	
H8B	1.068152	0.617707	0.275367	0.033*	
C9	0.8427 (6)	0.7491 (3)	0.2307 (2)	0.0290 (7)	
H9A	0.742172	0.735720	0.288698	0.035*	
H9B	0.866452	0.856524	0.223186	0.035*	
C10	0.7046 (6)	0.6407 (4)	0.1277 (2)	0.0283 (7)	

### 3,3',3'',3'''-{[Pyrazine-2,3,5,6-tetrayltetrakis(methylene))tetrakis(sulfanediyl]}tetrapropionic acid (H4L1A) Atomic displacement parameters (Å^2^)

**Table:**

	*U*^11^	*U*^22^	*U*^33^	*U*^12^	*U*^13^	*U*^23^
S1	0.0292 (5)	0.0246 (4)	0.0348 (4)	−0.0018 (3)	−0.0101 (3)	0.0109 (3)
S2	0.0243 (5)	0.0241 (4)	0.0376 (4)	−0.0013 (3)	0.0027 (3)	0.0156 (3)
O1	0.0333 (15)	0.0501 (14)	0.0362 (13)	−0.0022 (12)	−0.0093 (10)	0.0054 (11)
O2	0.0322 (16)	0.0486 (14)	0.0345 (13)	0.0001 (13)	−0.0099 (10)	0.0099 (10)
O3	0.0400 (15)	0.0425 (13)	0.0341 (12)	0.0216 (12)	−0.0002 (10)	0.0039 (10)
O4	0.0345 (15)	0.0519 (15)	0.0396 (13)	0.0217 (13)	−0.0064 (11)	0.0004 (12)
N1	0.0182 (14)	0.0159 (11)	0.0242 (11)	0.0010 (10)	−0.0009 (9)	0.0072 (9)
C1	0.0184 (16)	0.0158 (13)	0.0222 (13)	0.0006 (12)	0.0005 (10)	0.0055 (10)
C2	0.0182 (16)	0.0125 (12)	0.0226 (13)	0.0011 (12)	0.0009 (10)	0.0047 (10)
C3	0.0191 (17)	0.0189 (14)	0.0327 (15)	−0.0025 (13)	−0.0028 (11)	0.0085 (11)
C4	0.0259 (19)	0.0290 (16)	0.0359 (16)	−0.0069 (14)	−0.0053 (13)	0.0097 (13)
C5	0.032 (2)	0.0279 (16)	0.0404 (18)	0.0008 (15)	−0.0080 (14)	0.0114 (13)
C6	0.0228 (19)	0.0264 (16)	0.0360 (17)	0.0048 (15)	−0.0074 (13)	0.0083 (13)
C7	0.0251 (18)	0.0155 (13)	0.0313 (15)	0.0028 (13)	0.0021 (12)	0.0088 (11)
C8	0.0263 (18)	0.0316 (16)	0.0293 (15)	0.0092 (14)	0.0066 (12)	0.0132 (12)
C9	0.0281 (19)	0.0276 (16)	0.0324 (16)	0.0074 (15)	0.0008 (12)	0.0097 (12)
C10	0.0262 (18)	0.0385 (17)	0.0254 (15)	0.0125 (15)	0.0068 (12)	0.0129 (13)

### 3,3',3'',3'''-{[Pyrazine-2,3,5,6-tetrayltetrakis(methylene))tetrakis(sulfanediyl]}tetrapropionic acid (H4L1A) Geometric parameters (Å, º)

**Table:**

S1—C3	1.796 (3)	C3—H3B	0.9700
S1—C4	1.818 (3)	C4—C5	1.500 (4)
S2—C8	1.813 (3)	C4—H4A	0.9700
S2—C7	1.825 (3)	C4—H4B	0.9700
O1—C6	1.233 (4)	C5—C6	1.493 (4)
O2—C6	1.307 (4)	C5—H5A	0.9700
O2—H2O	0.87 (2)	C5—H5B	0.9700
O3—C10	1.240 (4)	C7—H7A	0.9700
O4—C10	1.294 (4)	C7—H7B	0.9700
O4—H4O	0.830 (19)	C8—C9	1.514 (4)
N1—C2	1.332 (3)	C8—H8A	0.9700
N1—C1	1.341 (3)	C8—H8B	0.9700
C1—C2^i^	1.406 (3)	C9—C10	1.499 (4)
C1—C3	1.499 (4)	C9—H9A	0.9700
C2—C7	1.500 (3)	C9—H9B	0.9700
C3—H3A	0.9700		
			
C3—S1—C4	97.53 (13)	C4—C5—H5B	108.8
C8—S2—C7	101.68 (14)	H5A—C5—H5B	107.7
C6—O2—H2O	108 (3)	O1—C6—O2	123.6 (3)
C10—O4—H4O	113 (3)	O1—C6—C5	122.7 (3)
C2—N1—C1	119.1 (2)	O2—C6—C5	113.7 (3)
N1—C1—C2^i^	120.3 (2)	C2—C7—S2	112.8 (2)
N1—C1—C3	117.7 (2)	C2—C7—H7A	109.0
C2^i^—C1—C3	122.0 (2)	S2—C7—H7A	109.0
N1—C2—C1^i^	120.6 (2)	C2—C7—H7B	109.0
N1—C2—C7	115.9 (2)	S2—C7—H7B	109.0
C1^i^—C2—C7	123.4 (2)	H7A—C7—H7B	107.8
C1—C3—S1	110.88 (18)	C9—C8—S2	114.2 (2)
C1—C3—H3A	109.5	C9—C8—H8A	108.7
S1—C3—H3A	109.5	S2—C8—H8A	108.7
C1—C3—H3B	109.5	C9—C8—H8B	108.7
S1—C3—H3B	109.5	S2—C8—H8B	108.7
H3A—C3—H3B	108.1	H8A—C8—H8B	107.6
C5—C4—S1	109.2 (2)	C10—C9—C8	113.5 (2)
C5—C4—H4A	109.8	C10—C9—H9A	108.9
S1—C4—H4A	109.8	C8—C9—H9A	108.9
C5—C4—H4B	109.8	C10—C9—H9B	108.9
S1—C4—H4B	109.8	C8—C9—H9B	108.9
H4A—C4—H4B	108.3	H9A—C9—H9B	107.7
C6—C5—C4	113.8 (3)	O3—C10—O4	122.8 (3)
C6—C5—H5A	108.8	O3—C10—C9	122.4 (3)
C4—C5—H5A	108.8	O4—C10—C9	114.8 (3)
C6—C5—H5B	108.8		
			
C2—N1—C1—C2^i^	−1.2 (4)	C4—C5—C6—O1	26.8 (4)
C2—N1—C1—C3	178.6 (2)	C4—C5—C6—O2	−154.4 (3)
C1—N1—C2—C1^i^	1.2 (4)	N1—C2—C7—S2	−94.0 (3)
C1—N1—C2—C7	179.6 (2)	C1^i^—C2—C7—S2	84.3 (3)
N1—C1—C3—S1	11.3 (3)	C8—S2—C7—C2	57.6 (2)
C2^i^—C1—C3—S1	−168.8 (2)	C7—S2—C8—C9	65.7 (2)
C4—S1—C3—C1	174.1 (2)	S2—C8—C9—C10	174.8 (2)
C3—S1—C4—C5	−155.3 (2)	C8—C9—C10—O3	8.8 (4)
S1—C4—C5—C6	−167.9 (2)	C8—C9—C10—O4	−171.8 (3)

Symmetry code: (i) −*x*+2, −*y*+1, −*z*+1.

### 3,3',3'',3'''-{[Pyrazine-2,3,5,6-tetrayltetrakis(methylene))tetrakis(sulfanediyl]}tetrapropionic acid (H4L1A) Hydrogen-bond geometry (Å, º)

**Table:**

*D*—H···*A*	*D*—H	H···*A*	*D*···*A*	*D*—H···*A*
O2—H2*O*···O1^ii^	0.87 (2)	1.80 (2)	2.667 (3)	172 (5)
O4—H4*O*···O3^iii^	0.83 (2)	1.85 (2)	2.673 (3)	175 (5)
C5—H5*A*···O3^iv^	0.97	2.55	3.405 (4)	147
C8—H8*A*···O4^v^	0.97	2.40	3.308 (4)	156

Symmetry codes: (ii) −*x*−1, −*y*, −*z*; (iii) −*x*+1, −*y*+1, −*z*; (iv) *x*−1, *y*, *z*; (v) *x*+1, *y*, *z*.

### 3,3',3'',3'''-{[Pyrazine-2,3,5,6-tetrayltetrakis(methylene))tetrakis(sulfanediyl]}tetrapropionic acid (H4L1B) Crystal data

**Table:**

C_20_H_28_N_2_O_8_S_4_	*Z* = 1
*M**_r_* = 552.68	*F*(000) = 290
Triclinic, *P*1	*D*_x_ = 1.509 Mg m^−^^3^
*a* = 4.9424 (17) Å	Mo *K*α radiation, λ = 0.71073 Å
*b* = 8.993 (3) Å	Cell parameters from 5563 reflections
*c* = 14.190 (6) Å	θ = 2.4–25.5°
α = 96.96 (3)°	µ = 0.44 mm^−^^1^
β = 97.14 (3)°	*T* = 293 K
γ = 100.72 (3)°	Plate, colourless
*V* = 608.1 (4) Å^3^	0.50 × 0.50 × 0.05 mm

### 3,3',3'',3'''-{[Pyrazine-2,3,5,6-tetrayltetrakis(methylene))tetrakis(sulfanediyl]}tetrapropionic acid (H4L1B) Data collection

**Table:**

STOE IPDS 2 diffractometer	2201 independent reflections
Radiation source: fine-focus sealed tube	1537 reflections with *I* > 2σ(*I*)
Plane graphite monochromator	*R*_int_ = 0.080
φ + ω scans	θ_max_ = 26.0°, θ_min_ = 2.3°
Absorption correction: empirical (using intensity measurements) (*ShxAbs*; Spek, 2020)	*h* = −5→5
*T*_min_ = 0.144, *T*_max_ = 0.616	*k* = −11→9
4152 measured reflections	*l* = −17→17

### 3,3',3'',3'''-{[Pyrazine-2,3,5,6-tetrayltetrakis(methylene))tetrakis(sulfanediyl]}tetrapropionic acid (H4L1B) Refinement

**Table:**

Refinement on *F*^2^	Primary atom site location: structure-invariant direct methods
Least-squares matrix: full	Secondary atom site location: difference Fourier map
*R*[*F*^2^ > 2σ(*F*^2^)] = 0.071	Hydrogen site location: inferred from neighbouring sites
*wR*(*F*^2^) = 0.208	H-atom parameters constrained
*S* = 1.05	*w* = 1/[σ^2^(*F*_o_^2^) + (0.1049*P*)^2^ + 0.4241*P*] where *P* = (*F*_o_^2^ + 2*F*_c_^2^)/3
2201 reflections	(Δ/σ)_max_ = 0.001
173 parameters	Δρ_max_ = 0.47 e Å^−^^3^
6 restraints	Δρ_min_ = −0.39 e Å^−^^3^

### 3,3',3'',3'''-{[Pyrazine-2,3,5,6-tetrayltetrakis(methylene))tetrakis(sulfanediyl]}tetrapropionic acid (H4L1B) Special details

**Table:**

Geometry. All esds (except the esd in the dihedral angle between two l.s. planes) are estimated using the full covariance matrix. The cell esds are taken into account individually in the estimation of esds in distances, angles and torsion angles; correlations between esds in cell parameters are only used when they are defined by crystal symmetry. An approximate (isotropic) treatment of cell esds is used for estimating esds involving l.s. planes.

### 3,3',3'',3'''-{[Pyrazine-2,3,5,6-tetrayltetrakis(methylene))tetrakis(sulfanediyl]}tetrapropionic acid (H4L1B) Fractional atomic coordinates and isotropic or equivalent isotropic displacement parameters (Å^2^)

**Table:**

	*x*	*y*	*z*	*U*_iso_*/*U*_eq_	Occ. (<1)
S1	0.7886 (3)	0.28919 (12)	0.71512 (9)	0.0583 (4)	
S2	0.6227 (3)	0.07568 (12)	0.39724 (10)	0.0682 (5)	
N1	1.2114 (8)	0.5110 (4)	0.5754 (3)	0.0500 (9)	
C1	0.9965 (9)	0.3908 (4)	0.5566 (3)	0.0467 (10)	
C2	0.7852 (9)	0.3796 (4)	0.4821 (3)	0.0469 (10)	
C3	1.0020 (10)	0.2707 (4)	0.6214 (3)	0.0537 (11)	
H3A	1.192760	0.277226	0.650755	0.064*	
H3B	0.937959	0.170470	0.583113	0.064*	
C4	0.9971 (12)	0.4560 (5)	0.7916 (4)	0.0644 (13)	
H4A	1.087916	0.524956	0.752231	0.077*	
H4B	0.876729	0.509027	0.826037	0.077*	
C5	1.2177 (12)	0.4158 (6)	0.8638 (4)	0.0682 (14)	
H5A	1.332589	0.358948	0.829232	0.082*	
H5B	1.337009	0.509683	0.898069	0.082*	
C6	1.0984 (14)	0.3238 (6)	0.9345 (4)	0.0728 (15)	
O1	0.8532 (10)	0.3158 (5)	0.9473 (3)	0.0828 (12)	
O2	1.2630 (12)	0.2603 (9)	0.9826 (5)	0.136 (2)	
H2O	1.173990	0.186783	1.001978	0.204*	
C7	0.5410 (10)	0.2468 (5)	0.4572 (4)	0.0559 (11)	
H7A	0.394220	0.276430	0.416093	0.067*	
H7B	0.470023	0.224151	0.515671	0.067*	
C8	0.6926 (16)	0.1494 (6)	0.2872 (5)	0.0604 (17)	0.821 (6)
H8A	0.530565	0.183752	0.258819	0.072*	0.821 (6)
H8B	0.847467	0.236521	0.301719	0.072*	0.821 (6)
C9	0.7607 (15)	0.0292 (6)	0.2173 (5)	0.0657 (16)	0.821 (6)
H9A	0.597449	−0.051856	0.196091	0.079*	0.821 (6)
H9B	0.906849	−0.014697	0.248293	0.079*	0.821 (6)
C10	0.8546 (16)	0.0963 (7)	0.1325 (5)	0.0622 (15)	0.821 (6)
O3	1.0881 (16)	0.0900 (11)	0.1127 (6)	0.132 (3)	0.821 (6)
O4	0.6974 (19)	0.1598 (10)	0.0858 (6)	0.117 (3)	0.821 (6)
H4O	0.785351	0.212989	0.052298	0.175*	0.821 (6)
C8B	0.834 (7)	0.112 (3)	0.3062 (18)	0.0604 (17)	0.179 (6)
H8B1	0.957385	0.212176	0.321614	0.072*	0.179 (6)
H8B2	0.941870	0.034250	0.294752	0.072*	0.179 (6)
C9B	0.609 (6)	0.106 (3)	0.2219 (19)	0.0657 (16)	0.179 (6)
H9B1	0.490282	0.005115	0.207464	0.079*	0.179 (6)
H9B2	0.494650	0.179949	0.237286	0.079*	0.179 (6)
C10B	0.748 (6)	0.143 (4)	0.137 (2)	0.0622 (15)	0.179 (6)
O3B	0.998 (6)	0.188 (6)	0.137 (3)	0.132 (3)	0.179 (6)
O4B	0.576 (9)	0.176 (6)	0.073 (3)	0.117 (3)	0.179 (6)
H4OB	0.660348	0.228397	0.038152	0.175*	0.179 (6)

### 3,3',3'',3'''-{[Pyrazine-2,3,5,6-tetrayltetrakis(methylene))tetrakis(sulfanediyl]}tetrapropionic acid (H4L1B) Atomic displacement parameters (Å^2^)

**Table:**

	*U*^11^	*U*^22^	*U*^33^	*U*^12^	*U*^13^	*U*^23^
S1	0.0546 (9)	0.0462 (6)	0.0762 (8)	0.0041 (5)	0.0158 (6)	0.0205 (5)
S2	0.0868 (11)	0.0329 (5)	0.0834 (9)	−0.0047 (5)	0.0293 (7)	0.0119 (5)
N1	0.045 (2)	0.0322 (16)	0.075 (2)	0.0074 (14)	0.0141 (17)	0.0146 (15)
C1	0.048 (3)	0.0259 (16)	0.071 (3)	0.0081 (15)	0.017 (2)	0.0160 (16)
C2	0.045 (3)	0.0292 (17)	0.070 (3)	0.0054 (15)	0.017 (2)	0.0152 (16)
C3	0.051 (3)	0.0350 (19)	0.080 (3)	0.0097 (17)	0.015 (2)	0.0220 (19)
C4	0.073 (4)	0.043 (2)	0.083 (3)	0.009 (2)	0.026 (3)	0.022 (2)
C5	0.063 (4)	0.061 (3)	0.077 (3)	−0.001 (2)	0.015 (3)	0.017 (2)
C6	0.064 (4)	0.066 (3)	0.090 (4)	0.003 (2)	0.013 (3)	0.033 (3)
O1	0.079 (3)	0.086 (3)	0.093 (3)	0.019 (2)	0.029 (2)	0.034 (2)
O2	0.085 (4)	0.190 (6)	0.162 (5)	0.031 (4)	0.031 (3)	0.122 (5)
C7	0.051 (3)	0.042 (2)	0.073 (3)	−0.0032 (18)	0.012 (2)	0.0155 (19)
C8	0.078 (5)	0.034 (3)	0.074 (4)	0.010 (2)	0.023 (3)	0.017 (2)
C9	0.084 (5)	0.040 (3)	0.079 (4)	0.011 (3)	0.026 (3)	0.022 (3)
C10	0.068 (5)	0.048 (3)	0.080 (4)	0.019 (3)	0.024 (3)	0.020 (3)
O3	0.096 (5)	0.194 (8)	0.145 (6)	0.052 (5)	0.052 (4)	0.113 (6)
O4	0.132 (8)	0.140 (5)	0.131 (5)	0.087 (5)	0.067 (5)	0.090 (4)
C8B	0.078 (5)	0.034 (3)	0.074 (4)	0.010 (2)	0.023 (3)	0.017 (2)
C9B	0.084 (5)	0.040 (3)	0.079 (4)	0.011 (3)	0.026 (3)	0.022 (3)
C10B	0.068 (5)	0.048 (3)	0.080 (4)	0.019 (3)	0.024 (3)	0.020 (3)
O3B	0.096 (5)	0.194 (8)	0.145 (6)	0.052 (5)	0.052 (4)	0.113 (6)
O4B	0.132 (8)	0.140 (5)	0.131 (5)	0.087 (5)	0.067 (5)	0.090 (4)

### 3,3',3'',3'''-{[Pyrazine-2,3,5,6-tetrayltetrakis(methylene))tetrakis(sulfanediyl]}tetrapropionic acid (H4L1B) Geometric parameters (Å, º)

**Table:**

S1—C4	1.801 (5)	C7—H7A	0.9700
S1—C3	1.808 (5)	C7—H7B	0.9700
S2—C8B	1.78 (3)	C8—C9	1.490 (8)
S2—C7	1.804 (5)	C8—H8A	0.9700
S2—C8	1.816 (6)	C8—H8B	0.9700
N1—C1	1.342 (5)	C9—C10	1.496 (8)
N1—C2^i^	1.351 (5)	C9—H9A	0.9700
C1—C2	1.371 (6)	C9—H9B	0.9700
C1—C3	1.503 (5)	C10—O4	1.224 (8)
C2—C7	1.504 (6)	C10—O3	1.231 (9)
C3—H3A	0.9700	O4—H4O	0.8200
C3—H3B	0.9700	C8B—C9B	1.516 (19)
C4—C5	1.526 (8)	C8B—H8B1	0.9700
C4—H4A	0.9700	C8B—H8B2	0.9700
C4—H4B	0.9700	C9B—C10B	1.505 (19)
C5—C6	1.485 (7)	C9B—H9B1	0.9700
C5—H5A	0.9700	C9B—H9B2	0.9700
C5—H5B	0.9700	C10B—O3B	1.226 (19)
C6—O1	1.238 (7)	C10B—O4B	1.265 (19)
C6—O2	1.258 (8)	O4B—H4OB	0.8200
O2—H2O	0.8200		
			
C4—S1—C3	100.2 (2)	C2—C7—H7B	108.8
C8B—S2—C7	112.9 (9)	S2—C7—H7B	108.8
C7—S2—C8	96.8 (2)	H7A—C7—H7B	107.7
C1—N1—C2^i^	117.9 (4)	C9—C8—S2	110.8 (4)
N1—C1—C2	121.2 (4)	C9—C8—H8A	109.5
N1—C1—C3	116.3 (4)	S2—C8—H8A	109.5
C2—C1—C3	122.5 (4)	C9—C8—H8B	109.5
N1^i^—C2—C1	120.9 (4)	S2—C8—H8B	109.5
N1^i^—C2—C7	115.7 (4)	H8A—C8—H8B	108.1
C1—C2—C7	123.5 (4)	C8—C9—C10	110.3 (5)
C1—C3—S1	113.3 (3)	C8—C9—H9A	109.6
C1—C3—H3A	108.9	C10—C9—H9A	109.6
S1—C3—H3A	108.9	C8—C9—H9B	109.6
C1—C3—H3B	108.9	C10—C9—H9B	109.6
S1—C3—H3B	108.9	H9A—C9—H9B	108.1
H3A—C3—H3B	107.7	O4—C10—O3	121.6 (7)
C5—C4—S1	112.3 (3)	O4—C10—C9	118.5 (7)
C5—C4—H4A	109.2	O3—C10—C9	119.8 (6)
S1—C4—H4A	109.2	C10—O4—H4O	109.5
C5—C4—H4B	109.2	C9B—C8B—S2	99.9 (19)
S1—C4—H4B	109.2	C9B—C8B—H8B1	111.8
H4A—C4—H4B	107.9	S2—C8B—H8B1	111.8
C6—C5—C4	113.3 (5)	C9B—C8B—H8B2	111.8
C6—C5—H5A	108.9	S2—C8B—H8B2	111.8
C4—C5—H5A	108.9	H8B1—C8B—H8B2	109.5
C6—C5—H5B	108.9	C10B—C9B—C8B	108 (2)
C4—C5—H5B	108.9	C10B—C9B—H9B1	110.0
H5A—C5—H5B	107.7	C8B—C9B—H9B1	110.0
O1—C6—O2	122.4 (5)	C10B—C9B—H9B2	110.0
O1—C6—C5	121.3 (5)	C8B—C9B—H9B2	110.0
O2—C6—C5	116.3 (6)	H9B1—C9B—H9B2	108.4
C6—O2—H2O	109.5	O3B—C10B—O4B	119 (3)
C2—C7—S2	113.8 (3)	O3B—C10B—C9B	126 (3)
C2—C7—H7A	108.8	O4B—C10B—C9B	110 (3)
S2—C7—H7A	108.8	C10B—O4B—H4OB	109.5
			
C2^i^—N1—C1—C2	−0.6 (6)	N1^i^—C2—C7—S2	103.4 (4)
C2^i^—N1—C1—C3	179.3 (4)	C1—C2—C7—S2	−75.4 (5)
N1—C1—C2—N1^i^	0.6 (7)	C8B—S2—C7—C2	−43.7 (10)
C3—C1—C2—N1^i^	−179.3 (4)	C8—S2—C7—C2	−66.8 (4)
N1—C1—C2—C7	179.3 (4)	C7—S2—C8—C9	−178.1 (5)
C3—C1—C2—C7	−0.6 (6)	S2—C8—C9—C10	−172.5 (5)
N1—C1—C3—S1	98.8 (4)	C8—C9—C10—O4	−57.8 (10)
C2—C1—C3—S1	−81.3 (5)	C8—C9—C10—O3	120.5 (9)
C4—S1—C3—C1	−72.6 (4)	C7—S2—C8B—C9B	−87.0 (16)
C3—S1—C4—C5	−86.7 (4)	S2—C8B—C9B—C10B	177 (2)
S1—C4—C5—C6	−65.0 (6)	C8B—C9B—C10B—O3B	−8 (5)
C4—C5—C6—O1	−17.0 (8)	C8B—C9B—C10B—O4B	−164 (3)
C4—C5—C6—O2	165.7 (6)		

Symmetry code: (i) −*x*+2, −*y*+1, −*z*+1.

### 3,3',3'',3'''-{[Pyrazine-2,3,5,6-tetrayltetrakis(methylene))tetrakis(sulfanediyl]}tetrapropionic acid (H4L1B) Hydrogen-bond geometry (Å, º)

**Table:**

*D*—H···*A*	*D*—H	H···*A*	*D*···*A*	*D*—H···*A*
O2—H2*O*···O3^ii^	0.82	1.94	2.66 (1)	146
O2—H2*O*···O3*B*^ii^	0.82	2.20	2.77 (3)	127
O4—H4*O*···O1^iii^	0.82	1.88	2.66 (1)	158
O4*B*—H4*OB*···O1^iii^	0.82	1.86	2.67 (4)	170

Symmetry codes: (ii) *x*, *y*, *z*+1; (iii) *x*, *y*, *z*−1.

### Poly[(µ-3-{[(3,5,6-tris{[(2-carboxyethyl)sulfanyl]methyl}pyrazin-2-yl)methyl]sulfanyl}propanoato)potassium] (KH3L1) Crystal data

**Table:**

[K(C_20_H_27_N_2_O_8_S_4_)]	*F*(000) = 1232
*M**_r_* = 590.77	*D*_x_ = 1.611 Mg m^−^^3^
Monoclinic, *C*2/*c*	Mo *K*α radiation, λ = 0.71073 Å
*a* = 30.080 (4) Å	Cell parameters from 7965 reflections
*b* = 8.4716 (10) Å	θ = 1.4–25.0°
*c* = 9.5908 (12) Å	µ = 0.61 mm^−^^1^
β = 94.717 (11)°	*T* = 153 K
*V* = 2435.7 (6) Å^3^	Plate, colourless
*Z* = 4	0.50 × 0.50 × 0.10 mm

### Poly[(µ-3-{[(3,5,6-tris{[(2-carboxyethyl)sulfanyl]methyl}pyrazin-2-yl)methyl]sulfanyl}propanoato)potassium] (KH3L1) Data collection

**Table:**

STOE IPDS 2 diffractometer	2084 independent reflections
Radiation source: fine-focus sealed tube	1646 reflections with *I* > 2σ(*I*)
Plane graphite monochromator	*R*_int_ = 0.064
φ + ω scans	θ_max_ = 24.8°, θ_min_ = 2.5°
Absorption correction: multi-scan (MULABS; Spek, 2020)	*h* = −35→35
*T*_min_ = 0.640, *T*_max_ = 1.000	*k* = −9→9
10309 measured reflections	*l* = −11→11

### Poly[(µ-3-{[(3,5,6-tris{[(2-carboxyethyl)sulfanyl]methyl}pyrazin-2-yl)methyl]sulfanyl}propanoato)potassium] (KH3L1) Refinement

**Table:**

Refinement on *F*^2^	Primary atom site location: structure-invariant direct methods
Least-squares matrix: full	Secondary atom site location: difference Fourier map
*R*[*F*^2^ > 2σ(*F*^2^)] = 0.039	Hydrogen site location: mixed
*wR*(*F*^2^) = 0.106	H atoms treated by a mixture of independent and constrained refinement
*S* = 1.02	*w* = 1/[σ^2^(*F*_o_^2^) + (0.0648*P*)^2^ + 1.5958*P*] where *P* = (*F*_o_^2^ + 2*F*_c_^2^)/3
2084 reflections	(Δ/σ)_max_ = 0.002
165 parameters	Δρ_max_ = 0.26 e Å^−^^3^
0 restraints	Δρ_min_ = −0.36 e Å^−^^3^

### Poly[(µ-3-{[(3,5,6-tris{[(2-carboxyethyl)sulfanyl]methyl}pyrazin-2-yl)methyl]sulfanyl}propanoato)potassium] (KH3L1) Special details

**Table:**

Geometry. All esds (except the esd in the dihedral angle between two l.s. planes) are estimated using the full covariance matrix. The cell esds are taken into account individually in the estimation of esds in distances, angles and torsion angles; correlations between esds in cell parameters are only used when they are defined by crystal symmetry. An approximate (isotropic) treatment of cell esds is used for estimating esds involving l.s. planes.

### Poly[(µ-3-{[(3,5,6-tris{[(2-carboxyethyl)sulfanyl]methyl}pyrazin-2-yl)methyl]sulfanyl}propanoato)potassium] (KH3L1) Fractional atomic coordinates and isotropic or equivalent isotropic displacement parameters (Å^2^)

**Table:**

	*x*	*y*	*z*	*U*_iso_*/*U*_eq_	
K1	0.500000	−0.28423 (13)	−0.250000	0.0357 (3)	
S1	0.34409 (2)	−0.03261 (9)	0.17646 (7)	0.0305 (2)	
S2	0.18978 (2)	−0.11089 (9)	0.16606 (8)	0.0342 (2)	
O1	0.46781 (7)	−0.2235 (3)	0.0136 (2)	0.0404 (5)	
O2	0.46330 (7)	−0.0466 (3)	0.1860 (2)	0.0402 (6)	
H20	0.500000	−0.073 (9)	0.250000	0.12 (3)*	
O3	0.06245 (6)	0.1153 (3)	−0.0544 (2)	0.0363 (5)	
O4	0.03178 (7)	0.1057 (3)	0.1493 (2)	0.0472 (7)	
H4O	0.0125 (17)	0.157 (7)	0.109 (5)	0.097 (19)*	
N1	0.28457 (7)	0.2024 (3)	−0.0774 (2)	0.0240 (5)	
C1	0.27160 (8)	0.1117 (3)	0.0267 (3)	0.0240 (6)	
C2	0.23661 (8)	0.1606 (3)	0.1047 (3)	0.0229 (6)	
C3	0.29578 (8)	−0.0422 (3)	0.0500 (3)	0.0280 (6)	
H3A	0.305554	−0.079313	−0.040443	0.034*	
H3B	0.274689	−0.121452	0.082227	0.034*	
C4	0.38202 (9)	0.0765 (4)	0.0748 (3)	0.0352 (7)	
H4A	0.364384	0.148969	0.010857	0.042*	
H4B	0.401718	0.141946	0.139218	0.042*	
C5	0.41100 (9)	−0.0265 (4)	−0.0116 (3)	0.0335 (7)	
H5A	0.391756	−0.108161	−0.059355	0.040*	
H5B	0.422744	0.040143	−0.084955	0.040*	
C6	0.44979 (9)	−0.1079 (4)	0.0676 (3)	0.0326 (7)	
C7	0.22055 (9)	0.0650 (3)	0.2220 (3)	0.0283 (6)	
H7A	0.201211	0.132352	0.275893	0.034*	
H7B	0.246620	0.033583	0.285664	0.034*	
C8	0.14217 (9)	−0.0295 (4)	0.0628 (3)	0.0329 (7)	
H8A	0.152427	0.055219	0.002031	0.039*	
H8B	0.128312	−0.113189	0.001607	0.039*	
C9	0.10744 (9)	0.0371 (4)	0.1528 (3)	0.0350 (7)	
H9A	0.099826	−0.044379	0.220895	0.042*	
H9B	0.120475	0.128185	0.206358	0.042*	
C10	0.06542 (9)	0.0895 (4)	0.0702 (3)	0.0307 (7)	

### Poly[(µ-3-{[(3,5,6-tris{[(2-carboxyethyl)sulfanyl]methyl}pyrazin-2-yl)methyl]sulfanyl}propanoato)potassium] (KH3L1) Atomic displacement parameters (Å^2^)

**Table:**

	*U*^11^	*U*^22^	*U*^33^	*U*^12^	*U*^13^	*U*^23^
K1	0.0254 (4)	0.0547 (6)	0.0267 (5)	0.000	−0.0009 (3)	0.000
S1	0.0189 (3)	0.0428 (5)	0.0297 (4)	0.0034 (3)	0.0011 (3)	0.0042 (3)
S2	0.0224 (4)	0.0335 (4)	0.0466 (5)	−0.0003 (3)	0.0027 (3)	0.0034 (3)
O1	0.0302 (11)	0.0590 (15)	0.0320 (11)	0.0130 (10)	0.0022 (9)	−0.0023 (10)
O2	0.0246 (10)	0.0633 (15)	0.0319 (11)	0.0051 (10)	−0.0024 (9)	−0.0077 (10)
O3	0.0259 (10)	0.0554 (15)	0.0278 (11)	0.0021 (9)	0.0032 (8)	0.0038 (9)
O4	0.0266 (12)	0.0804 (19)	0.0356 (13)	0.0138 (12)	0.0085 (10)	0.0044 (12)
N1	0.0156 (10)	0.0304 (13)	0.0255 (12)	−0.0007 (9)	−0.0019 (9)	−0.0021 (10)
C1	0.0152 (12)	0.0299 (16)	0.0256 (14)	0.0007 (11)	−0.0048 (10)	−0.0038 (11)
C2	0.0159 (11)	0.0278 (15)	0.0239 (14)	−0.0009 (11)	−0.0036 (10)	−0.0026 (11)
C3	0.0187 (13)	0.0317 (16)	0.0331 (15)	0.0022 (11)	−0.0012 (11)	−0.0003 (12)
C4	0.0210 (13)	0.0424 (19)	0.0421 (18)	−0.0024 (13)	0.0018 (12)	0.0070 (14)
C5	0.0213 (13)	0.051 (2)	0.0280 (15)	−0.0009 (12)	0.0017 (12)	0.0039 (13)
C6	0.0185 (13)	0.053 (2)	0.0268 (15)	−0.0019 (13)	0.0047 (11)	0.0024 (14)
C7	0.0229 (14)	0.0366 (16)	0.0251 (14)	−0.0005 (12)	0.0013 (11)	0.0024 (12)
C8	0.0224 (14)	0.0422 (19)	0.0338 (16)	−0.0034 (12)	0.0011 (12)	−0.0009 (13)
C9	0.0208 (14)	0.056 (2)	0.0280 (15)	0.0017 (13)	0.0037 (12)	0.0032 (14)
C10	0.0217 (13)	0.0388 (18)	0.0321 (16)	−0.0033 (12)	0.0052 (12)	−0.0013 (13)

### Poly[(µ-3-{[(3,5,6-tris{[(2-carboxyethyl)sulfanyl]methyl}pyrazin-2-yl)methyl]sulfanyl}propanoato)potassium] (KH3L1) Geometric parameters (Å, º)

**Table:**

K1—O1	2.828 (2)	C1—C2	1.403 (4)
K1—O1^i^	2.828 (2)	C1—C3	1.501 (4)
K1—O2^ii^	3.056 (3)	C2—C7	1.498 (4)
K1—O2^iii^	3.056 (3)	C3—H3A	0.9900
K1—O3^iv^	2.682 (2)	C3—H3B	0.9900
K1—O3^v^	2.682 (2)	C4—C5	1.525 (4)
K1—O4^vi^	3.069 (3)	C4—H4A	0.9900
K1—O4^vii^	3.069 (3)	C4—H4B	0.9900
S1—C4	1.814 (3)	C5—C6	1.506 (4)
S1—C3	1.816 (3)	C5—H5A	0.9900
S2—C8	1.809 (3)	C5—H5B	0.9900
S2—C7	1.812 (3)	C7—H7A	0.9900
O1—C6	1.252 (4)	C7—H7B	0.9900
O2—C6	1.284 (4)	C8—C9	1.517 (4)
O2—H20	1.239 (15)	C8—H8A	0.9900
O3—C10	1.211 (3)	C8—H8B	0.9900
O4—C10	1.321 (3)	C9—C10	1.502 (4)
O4—H4O	0.80 (5)	C9—H9A	0.9900
N1—C2^viii^	1.339 (4)	C9—H9B	0.9900
N1—C1	1.343 (4)		
			
O3^iv^—K1—O3^v^	143.00 (11)	C1—C2—C7	122.9 (3)
O3^iv^—K1—O1	114.30 (6)	C1—C3—S1	114.31 (19)
O3^v^—K1—O1	72.78 (6)	C1—C3—H3A	108.7
O3^iv^—K1—O1^i^	72.78 (6)	S1—C3—H3A	108.7
O3^v^—K1—O1^i^	114.30 (6)	C1—C3—H3B	108.7
O1—K1—O1^i^	159.05 (11)	S1—C3—H3B	108.7
O3^iv^—K1—O2^ii^	130.71 (7)	H3A—C3—H3B	107.6
O3^v^—K1—O2^ii^	85.98 (6)	C5—C4—S1	114.4 (2)
O1—K1—O2^ii^	78.37 (7)	C5—C4—H4A	108.7
O1^i^—K1—O2^ii^	82.42 (6)	S1—C4—H4A	108.7
O3^iv^—K1—O2^iii^	85.98 (6)	C5—C4—H4B	108.7
O3^v^—K1—O2^iii^	130.71 (7)	S1—C4—H4B	108.7
O1—K1—O2^iii^	82.42 (6)	H4A—C4—H4B	107.6
O1^i^—K1—O2^iii^	78.37 (7)	C6—C5—C4	116.2 (2)
O2^ii^—K1—O2^iii^	46.99 (8)	C6—C5—H5A	108.2
O3^iv^—K1—O4^vi^	73.54 (7)	C4—C5—H5A	108.2
O3^v^—K1—O4^vi^	73.75 (7)	C6—C5—H5B	108.2
O1—K1—O4^vi^	125.54 (7)	C4—C5—H5B	108.2
O1^i^—K1—O4^vi^	75.02 (7)	H5A—C5—H5B	107.4
O2^ii^—K1—O4^vi^	139.50 (6)	O1—C6—O2	124.5 (3)
O2^iii^—K1—O4^vi^	150.17 (6)	O1—C6—C5	119.6 (3)
O3^iv^—K1—O4^vii^	73.75 (7)	O2—C6—C5	115.9 (3)
O3^v^—K1—O4^vii^	73.54 (7)	C2—C7—S2	114.24 (19)
O1—K1—O4^vii^	75.02 (7)	C2—C7—H7A	108.7
O1^i^—K1—O4^vii^	125.54 (7)	S2—C7—H7A	108.7
O2^ii^—K1—O4^vii^	150.17 (6)	C2—C7—H7B	108.7
O2^iii^—K1—O4^vii^	139.50 (6)	S2—C7—H7B	108.7
O4^vi^—K1—O4^vii^	54.87 (9)	H7A—C7—H7B	107.6
C4—S1—C3	99.68 (14)	C9—C8—S2	112.4 (2)
C8—S2—C7	102.17 (14)	C9—C8—H8A	109.1
C6—O1—K1	134.73 (19)	S2—C8—H8A	109.1
C6—O2—K1^ii^	129.17 (19)	C9—C8—H8B	109.1
C6—O2—H20	125 (2)	S2—C8—H8B	109.1
K1^ii^—O2—H20	77 (4)	H8A—C8—H8B	107.9
C10—O3—K1^ix^	137.58 (18)	C10—C9—C8	113.5 (2)
C10—O4—K1^vii^	111.2 (2)	C10—C9—H9A	108.9
C10—O4—H4O	110 (4)	C8—C9—H9A	108.9
K1^vii^—O4—H4O	114 (4)	C10—C9—H9B	108.9
C2^viii^—N1—C1	118.6 (2)	C8—C9—H9B	108.9
N1—C1—C2	120.3 (2)	H9A—C9—H9B	107.7
N1—C1—C3	116.2 (2)	O3—C10—O4	123.4 (3)
C2—C1—C3	123.5 (2)	O3—C10—C9	124.3 (2)
N1^viii^—C2—C1	121.1 (2)	O4—C10—C9	112.3 (2)
N1^viii^—C2—C7	116.0 (2)		
			
C2^viii^—N1—C1—C2	−0.1 (4)	K1^ii^—O2—C6—C5	−60.6 (3)
C2^viii^—N1—C1—C3	178.5 (2)	C4—C5—C6—O1	161.6 (3)
N1—C1—C2—N1^viii^	0.1 (4)	C4—C5—C6—O2	−21.4 (4)
C3—C1—C2—N1^viii^	−178.4 (2)	N1^viii^—C2—C7—S2	107.9 (2)
N1—C1—C2—C7	180.0 (2)	C1—C2—C7—S2	−72.0 (3)
C3—C1—C2—C7	1.5 (4)	C8—S2—C7—C2	−62.3 (2)
N1—C1—C3—S1	91.6 (3)	C7—S2—C8—C9	−77.5 (2)
C2—C1—C3—S1	−89.8 (3)	S2—C8—C9—C10	−173.8 (2)
C4—S1—C3—C1	−72.3 (2)	K1^ix^—O3—C10—O4	1.7 (5)
C3—S1—C4—C5	−90.3 (2)	K1^ix^—O3—C10—C9	−177.7 (2)
S1—C4—C5—C6	−76.4 (3)	K1^vii^—O4—C10—O3	110.5 (3)
K1—O1—C6—O2	−127.8 (3)	K1^vii^—O4—C10—C9	−70.0 (3)
K1—O1—C6—C5	48.8 (4)	C8—C9—C10—O3	−17.9 (5)
K1^ii^—O2—C6—O1	116.1 (3)	C8—C9—C10—O4	162.6 (3)

Symmetry codes: (i) −*x*+1, *y*, −*z*−1/2; (ii) −*x*+1, −*y*, −*z*; (iii) *x*, −*y*, *z*−1/2; (iv) −*x*+1/2, *y*−1/2, −*z*−1/2; (v) *x*+1/2, *y*−1/2, *z*; (vi) *x*+1/2, −*y*−1/2, *z*−1/2; (vii) −*x*+1/2, −*y*−1/2, −*z*; (viii) −*x*+1/2, −*y*+1/2, −*z*; (ix) *x*−1/2, *y*+1/2, *z*.

### Poly[(µ-3-{[(3,5,6-tris{[(2-carboxyethyl)sulfanyl]methyl}pyrazin-2-yl)methyl]sulfanyl}propanoato)potassium] (KH3L1) Hydrogen-bond geometry (Å, º)

**Table:**

*D*—H···*A*	*D*—H	H···*A*	*D*···*A*	*D*—H···*A*
O4—H4*O*···O1^ix^	0.80 (5)	1.86 (5)	2.661 (3)	180 (7)
O2—H20···O2^x^	1.24 (1)	1.24 (1)	2.436 (3)	159 (7)
C4—H4*A*···N1	0.99	2.52	3.340 (4)	140
C4—H4*B*···O3^viii^	0.99	2.49	3.114 (4)	121
C5—H5*B*···O2^iii^	0.99	2.60	3.467 (4)	146
C7—H7*B*···N1^xi^	0.99	2.60	3.454 (4)	144
C9—H9*A*···O3^xi^	0.99	2.58	3.465 (4)	149

Symmetry codes: (iii) *x*, −*y*, *z*−1/2; (viii) −*x*+1/2, −*y*+1/2, −*z*; (ix) *x*−1/2, *y*+1/2, *z*; (x) −*x*+1, *y*, −*z*+1/2; (xi) *x*, −*y*, *z*+1/2.

### Poly[(µ-3,3'-{[(3,6-bis{[(2-carboxyethyl)sulfanyl]methyl}pyrazine-2,5-diyl)bis(methylene)]bis(sulfanediyl)}dipropionato)dipotassium] (K2H2L1) Crystal data

**Table:**

[K_2_(C_20_H_26_N_2_O_8_S_4_)]	*F*(000) = 1304
*M**_r_* = 628.87	*D*_x_ = 1.602 Mg m^−^^3^
Monoclinic, *C*2/*c*	Mo *K*α radiation, λ = 0.71073 Å
*a* = 27.908 (2) Å	Cell parameters from 20250 reflections
*b* = 8.2916 (6) Å	θ = 1.8–29.6°
*c* = 11.3035 (9) Å	µ = 0.73 mm^−^^1^
β = 94.753 (6)°	*T* = 153 K
*V* = 2606.7 (3) Å^3^	Plate, colourless
*Z* = 4	0.50 × 0.50 × 0.05 mm

### Poly[(µ-3,3'-{[(3,6-bis{[(2-carboxyethyl)sulfanyl]methyl}pyrazine-2,5-diyl)bis(methylene)]bis(sulfanediyl)}dipropionato)dipotassium] (K2H2L1) Data collection

**Table:**

STOE IPDS 2 diffractometer	3646 independent reflections
Radiation source: fine-focus sealed tube	3175 reflections with *I* > 2σ(*I*)
Plane graphite monochromator	*R*_int_ = 0.042
φ + ω scans	θ_max_ = 29.6°, θ_min_ = 2.6°
Absorption correction: empirical (using intensity measurements) (*ShxAbs*; Spek, 2020)	*h* = −38→38
*T*_min_ = 0.416, *T*_max_ = 0.803	*k* = −11→11
19423 measured reflections	*l* = −15→15

### Poly[(µ-3,3'-{[(3,6-bis{[(2-carboxyethyl)sulfanyl]methyl}pyrazine-2,5-diyl)bis(methylene)]bis(sulfanediyl)}dipropionato)dipotassium] (K2H2L1) Refinement

**Table:**

Refinement on *F*^2^	Primary atom site location: structure-invariant direct methods
Least-squares matrix: full	Secondary atom site location: difference Fourier map
*R*[*F*^2^ > 2σ(*F*^2^)] = 0.037	Hydrogen site location: mixed
*wR*(*F*^2^) = 0.103	H atoms treated by a mixture of independent and constrained refinement
*S* = 1.05	*w* = 1/[σ^2^(*F*_o_^2^) + (0.0541*P*)^2^ + 3.5192*P*] where *P* = (*F*_o_^2^ + 2*F*_c_^2^)/3
3646 reflections	(Δ/σ)_max_ < 0.001
167 parameters	Δρ_max_ = 0.76 e Å^−^^3^
1 restraint	Δρ_min_ = −0.51 e Å^−^^3^

### Poly[(µ-3,3'-{[(3,6-bis{[(2-carboxyethyl)sulfanyl]methyl}pyrazine-2,5-diyl)bis(methylene)]bis(sulfanediyl)}dipropionato)dipotassium] (K2H2L1) Special details

**Table:**

Geometry. All esds (except the esd in the dihedral angle between two l.s. planes) are estimated using the full covariance matrix. The cell esds are taken into account individually in the estimation of esds in distances, angles and torsion angles; correlations between esds in cell parameters are only used when they are defined by crystal symmetry. An approximate (isotropic) treatment of cell esds is used for estimating esds involving l.s. planes.

### Poly[(µ-3,3'-{[(3,6-bis{[(2-carboxyethyl)sulfanyl]methyl}pyrazine-2,5-diyl)bis(methylene)]bis(sulfanediyl)}dipropionato)dipotassium] (K2H2L1) Fractional atomic coordinates and isotropic or equivalent isotropic displacement parameters (Å^2^)

**Table:**

	*x*	*y*	*z*	*U*_iso_*/*U*_eq_	
K1	0.000000	0.82906 (7)	0.750000	0.02908 (13)	
K2	0.000000	0.64758 (6)	0.250000	0.02585 (12)	
S1	0.15530 (2)	0.94369 (6)	0.36528 (5)	0.03200 (12)	
S2	0.31454 (2)	0.91861 (6)	0.30731 (4)	0.02972 (12)	
O1	0.03202 (5)	0.90961 (16)	0.37679 (12)	0.0288 (3)	
O2	0.03173 (4)	0.75961 (15)	0.53962 (10)	0.0251 (2)	
O3	0.44445 (5)	1.07891 (16)	0.64145 (11)	0.0264 (3)	
O4	0.45504 (4)	1.14483 (16)	0.45457 (11)	0.0246 (2)	
H4O	0.4811 (7)	1.187 (3)	0.485 (2)	0.037*	
N1	0.22122 (5)	1.18192 (18)	0.58094 (13)	0.0226 (3)	
C1	0.23166 (6)	1.10103 (19)	0.48370 (15)	0.0214 (3)	
C2	0.26072 (6)	1.1694 (2)	0.40166 (14)	0.0214 (3)	
C3	0.20952 (6)	0.9373 (2)	0.46641 (17)	0.0261 (3)	
H3A	0.201509	0.894439	0.544105	0.031*	
H3B	0.233073	0.863252	0.434300	0.031*	
C4	0.11775 (7)	1.0635 (2)	0.4543 (2)	0.0344 (4)	
H4A	0.138286	1.142471	0.500499	0.041*	
H4B	0.094632	1.125024	0.400576	0.041*	
C5	0.08989 (6)	0.9666 (2)	0.53952 (18)	0.0301 (4)	
H5A	0.112007	0.889825	0.583250	0.036*	
H5B	0.077277	1.040676	0.598231	0.036*	
C6	0.04848 (6)	0.8740 (2)	0.47738 (14)	0.0215 (3)	
C7	0.27238 (6)	1.0859 (2)	0.28997 (15)	0.0260 (3)	
H7A	0.285609	1.166910	0.237196	0.031*	
H7B	0.241998	1.045256	0.249183	0.031*	
C8	0.37036 (6)	1.0182 (3)	0.35882 (16)	0.0295 (4)	
H8A	0.397073	0.966995	0.320275	0.035*	
H8B	0.368597	1.132425	0.333262	0.035*	
C9	0.38174 (6)	1.0126 (2)	0.49202 (15)	0.0243 (3)	
H9A	0.380284	0.899037	0.518480	0.029*	
H9B	0.356583	1.073093	0.530128	0.029*	
C10	0.43036 (6)	1.08130 (19)	0.53492 (14)	0.0216 (3)	

### Poly[(µ-3,3'-{[(3,6-bis{[(2-carboxyethyl)sulfanyl]methyl}pyrazine-2,5-diyl)bis(methylene)]bis(sulfanediyl)}dipropionato)dipotassium] (K2H2L1) Atomic displacement parameters (Å^2^)

**Table:**

	*U*^11^	*U*^22^	*U*^33^	*U*^12^	*U*^13^	*U*^23^
K1	0.0442 (3)	0.0240 (2)	0.0186 (2)	0.000	−0.0001 (2)	0.000
K2	0.0356 (3)	0.0230 (2)	0.0189 (2)	0.000	0.00191 (18)	0.000
S1	0.0250 (2)	0.0304 (2)	0.0399 (3)	−0.00503 (16)	−0.00202 (17)	−0.00557 (18)
S2	0.0236 (2)	0.0297 (2)	0.0355 (2)	−0.00158 (16)	0.00042 (16)	−0.00746 (17)
O1	0.0311 (6)	0.0272 (6)	0.0269 (6)	−0.0064 (5)	−0.0040 (5)	0.0023 (5)
O2	0.0235 (5)	0.0282 (6)	0.0233 (5)	−0.0027 (5)	0.0009 (4)	0.0005 (5)
O3	0.0276 (6)	0.0300 (6)	0.0217 (5)	−0.0011 (5)	0.0019 (4)	0.0021 (5)
O4	0.0228 (5)	0.0299 (6)	0.0212 (5)	−0.0051 (5)	0.0024 (4)	0.0004 (5)
N1	0.0196 (6)	0.0235 (6)	0.0242 (6)	−0.0010 (5)	−0.0004 (5)	0.0030 (5)
C1	0.0173 (6)	0.0206 (7)	0.0256 (7)	−0.0002 (5)	−0.0027 (5)	0.0020 (6)
C2	0.0183 (7)	0.0230 (7)	0.0223 (7)	0.0002 (5)	−0.0022 (5)	0.0019 (6)
C3	0.0214 (7)	0.0213 (7)	0.0353 (9)	−0.0017 (6)	0.0007 (6)	0.0016 (6)
C4	0.0220 (8)	0.0231 (8)	0.0576 (12)	−0.0023 (6)	−0.0009 (8)	−0.0060 (8)
C5	0.0230 (8)	0.0295 (9)	0.0368 (9)	−0.0023 (6)	−0.0035 (7)	−0.0092 (7)
C6	0.0180 (7)	0.0212 (7)	0.0250 (7)	0.0004 (5)	0.0006 (5)	−0.0046 (6)
C7	0.0250 (8)	0.0290 (8)	0.0237 (7)	−0.0007 (6)	−0.0005 (6)	−0.0005 (6)
C8	0.0211 (7)	0.0402 (10)	0.0271 (8)	−0.0049 (7)	0.0021 (6)	−0.0020 (7)
C9	0.0218 (7)	0.0250 (8)	0.0261 (7)	−0.0017 (6)	0.0025 (6)	−0.0010 (6)
C10	0.0224 (7)	0.0205 (7)	0.0220 (7)	0.0019 (5)	0.0031 (6)	−0.0003 (5)

### Poly[(µ-3,3'-{[(3,6-bis{[(2-carboxyethyl)sulfanyl]methyl}pyrazine-2,5-diyl)bis(methylene)]bis(sulfanediyl)}dipropionato)dipotassium] (K2H2L1) Geometric parameters (Å, º)

**Table:**

K1—O1^i^	2.7084 (14)	O1—C6	1.227 (2)
K1—O1^ii^	2.7084 (14)	O4—C10	1.296 (2)
K1—O2	2.6682 (12)	O4—H4O	0.855 (16)
K1—O2^iii^	2.6683 (12)	O3—C10	1.236 (2)
K1—O3^iv^	2.8099 (14)	N1—C1	1.340 (2)
K1—O3^v^	2.8099 (13)	N1—C2^xi^	1.341 (2)
K1—C6^iii^	3.4864 (17)	C1—C2	1.401 (2)
K1—C6	3.4865 (17)	C1—C3	1.498 (2)
K1—K2^vi^	3.9521 (8)	C2—C7	1.499 (2)
K1—K2^ii^	4.3395 (8)	C3—H3A	0.9900
K2—O1^vii^	2.7131 (13)	C3—H3B	0.9900
K2—O1	2.7132 (13)	C4—C5	1.518 (3)
K2—O3^viii^	2.6683 (13)	C4—H4A	0.9900
K2—O3^ix^	2.6682 (13)	C4—H4B	0.9900
K2—O4^x^	2.7209 (12)	C5—C6	1.511 (2)
K2—O4^iv^	2.7209 (12)	C5—H5A	0.9900
K2—C6^vii^	3.3739 (16)	C5—H5B	0.9900
K2—C6	3.3739 (16)	C7—H7A	0.9900
K2—C10^viii^	3.5364 (16)	C7—H7B	0.9900
K2—C10^ix^	3.5364 (16)	C8—C9	1.513 (2)
S1—C4	1.809 (2)	C8—H8A	0.9900
S1—C3	1.8200 (18)	C8—H8B	0.9900
S2—C8	1.8159 (18)	C9—C10	1.514 (2)
S2—C7	1.8190 (19)	C9—H9A	0.9900
O2—C6	1.291 (2)	C9—H9B	0.9900
			
O2—K1—O2^iii^	155.07 (6)	O3^ix^—K2—K1^vi^	45.27 (3)
O2—K1—O1^i^	121.67 (4)	O1^vii^—K2—K1^vi^	143.21 (3)
O2^iii^—K1—O1^i^	79.65 (4)	O1—K2—K1^vi^	143.21 (3)
O2—K1—O1^ii^	79.65 (4)	O4^x^—K2—K1^vi^	89.52 (3)
O2^iii^—K1—O1^ii^	121.67 (4)	O4^iv^—K2—K1^vi^	89.52 (3)
O1^i^—K1—O1^ii^	73.73 (6)	C6^vii^—K2—K1^vi^	123.80 (3)
O2—K1—O3^iv^	70.32 (4)	C6—K2—K1^vi^	123.80 (3)
O2^iii^—K1—O3^iv^	91.03 (4)	C10^viii^—K2—K1^vi^	57.55 (3)
O1^i^—K1—O3^iv^	165.36 (4)	C10^ix^—K2—K1^vi^	57.55 (3)
O1^ii^—K1—O3^iv^	102.33 (4)	O3^viii^—K2—K1^ii^	134.73 (3)
O2—K1—O3^v^	91.03 (4)	O3^ix^—K2—K1^ii^	134.73 (3)
O2^iii^—K1—O3^v^	70.32 (4)	O1^vii^—K2—K1^ii^	36.79 (3)
O1^i^—K1—O3^v^	102.33 (4)	O1—K2—K1^ii^	36.79 (3)
O1^ii^—K1—O3^v^	165.36 (4)	O4^x^—K2—K1^ii^	90.48 (3)
O3^iv^—K1—O3^v^	84.85 (5)	O4^iv^—K2—K1^ii^	90.48 (3)
O2—K1—C6^iii^	172.97 (4)	C6^vii^—K2—K1^ii^	56.20 (3)
O2^iii^—K1—C6^iii^	18.84 (4)	C6—K2—K1^ii^	56.20 (3)
O1^i^—K1—C6^iii^	65.30 (4)	C10^viii^—K2—K1^ii^	122.45 (3)
O1^ii^—K1—C6^iii^	104.31 (4)	C10^ix^—K2—K1^ii^	122.45 (3)
O3^iv^—K1—C6^iii^	102.96 (4)	K1^vi^—K2—K1^ii^	180.0
O3^v^—K1—C6^iii^	86.17 (4)	C4—S1—C3	99.00 (9)
O2—K1—C6	18.84 (4)	C8—S2—C7	102.58 (9)
O2^iii^—K1—C6	172.97 (4)	C6—O2—K1	119.28 (10)
O1^i^—K1—C6	104.31 (4)	C6—O1—K1^ii^	139.92 (11)
O1^ii^—K1—C6	65.30 (4)	C6—O1—K2	112.22 (11)
O3^iv^—K1—C6	86.17 (4)	K1^ii^—O1—K2	106.34 (4)
O3^v^—K1—C6	102.96 (4)	C10—O4—K2^xii^	155.24 (11)
C6^iii^—K1—C6	167.74 (6)	C10—O4—H4O	111.3 (17)
O2—K1—K2^vi^	77.54 (3)	K2^xii^—O4—H4O	84.7 (17)
O2^iii^—K1—K2^vi^	77.54 (3)	C10—O3—K2^ix^	125.83 (11)
O1^i^—K1—K2^vi^	143.13 (3)	C10—O3—K1^xii^	122.23 (11)
O1^ii^—K1—K2^vi^	143.13 (3)	K2^ix^—O3—K1^xii^	92.31 (4)
O3^iv^—K1—K2^vi^	42.42 (3)	C1—N1—C2^xi^	118.43 (14)
O3^v^—K1—K2^vi^	42.42 (3)	N1—C1—C2	121.17 (15)
C6^iii^—K1—K2^vi^	96.13 (3)	N1—C1—C3	116.44 (15)
C6—K1—K2^vi^	96.13 (3)	C2—C1—C3	122.37 (15)
O2—K1—K2^ii^	102.46 (3)	N1^xi^—C2—C1	120.40 (15)
O2^iii^—K1—K2^ii^	102.46 (3)	N1^xi^—C2—C7	116.30 (15)
O1^i^—K1—K2^ii^	36.87 (3)	C1—C2—C7	123.28 (15)
O1^ii^—K1—K2^ii^	36.87 (3)	C1—C3—S1	111.60 (12)
O3^iv^—K1—K2^ii^	137.58 (3)	C1—C3—H3A	109.3
O3^v^—K1—K2^ii^	137.58 (3)	S1—C3—H3A	109.3
C6^iii^—K1—K2^ii^	83.87 (3)	C1—C3—H3B	109.3
C6—K1—K2^ii^	83.87 (3)	S1—C3—H3B	109.3
K2^vi^—K1—K2^ii^	180.0	H3A—C3—H3B	108.0
O3^viii^—K2—O3^ix^	90.54 (6)	C5—C4—S1	114.41 (14)
O3^viii^—K2—O1^vii^	99.63 (4)	C5—C4—H4A	108.7
O3^ix^—K2—O1^vii^	163.72 (4)	S1—C4—H4A	108.7
O3^viii^—K2—O1	163.72 (4)	C5—C4—H4B	108.7
O3^ix^—K2—O1	99.63 (4)	S1—C4—H4B	108.7
O1^vii^—K2—O1	73.59 (6)	H4A—C4—H4B	107.6
O3^viii^—K2—O4^x^	83.95 (4)	C6—C5—C4	112.73 (16)
O3^ix^—K2—O4^x^	95.38 (4)	C6—C5—H5A	109.0
O1^vii^—K2—O4^x^	73.30 (4)	C4—C5—H5A	109.0
O1—K2—O4^x^	107.50 (4)	C6—C5—H5B	109.0
O3^viii^—K2—O4^iv^	95.38 (4)	C4—C5—H5B	109.0
O3^ix^—K2—O4^iv^	83.95 (4)	H5A—C5—H5B	107.8
O1^vii^—K2—O4^iv^	107.50 (4)	O1—C6—O2	123.88 (15)
O1—K2—O4^iv^	73.30 (4)	O1—C6—C5	121.42 (16)
O4^x^—K2—O4^iv^	179.04 (6)	O2—C6—C5	114.67 (15)
O3^viii^—K2—C6^vii^	81.96 (4)	O1—C6—K2	48.11 (8)
O3^ix^—K2—C6^vii^	157.35 (4)	O2—C6—K2	82.31 (9)
O1^vii^—K2—C6^vii^	19.67 (4)	C5—C6—K2	151.60 (11)
O1—K2—C6^vii^	92.90 (4)	O1—C6—K1	134.60 (11)
O4^x^—K2—C6^vii^	62.69 (4)	O2—C6—K1	41.88 (8)
O4^iv^—K2—C6^vii^	117.91 (4)	C5—C6—K1	88.93 (10)
O3^viii^—K2—C6	157.35 (4)	K2—C6—K1	116.96 (5)
O3^ix^—K2—C6	81.96 (4)	C2—C7—S2	116.42 (12)
O1^vii^—K2—C6	92.90 (4)	C2—C7—H7A	108.2
O1—K2—C6	19.67 (4)	S2—C7—H7A	108.2
O4^x^—K2—C6	117.91 (4)	C2—C7—H7B	108.2
O4^iv^—K2—C6	62.69 (4)	S2—C7—H7B	108.2
C6^vii^—K2—C6	112.40 (6)	H7A—C7—H7B	107.3
O3^viii^—K2—C10^viii^	16.46 (4)	C9—C8—S2	114.13 (13)
O3^ix^—K2—C10^viii^	101.75 (4)	C9—C8—H8A	108.7
O1^vii^—K2—C10^viii^	85.89 (4)	S2—C8—H8A	108.7
O1—K2—C10^viii^	158.61 (4)	C9—C8—H8B	108.7
O4^x^—K2—C10^viii^	71.16 (4)	S2—C8—H8B	108.7
O4^iv^—K2—C10^viii^	108.29 (4)	H8A—C8—H8B	107.6
C6^vii^—K2—C10^viii^	67.17 (4)	C8—C9—C10	114.57 (14)
C6—K2—C10^viii^	170.09 (4)	C8—C9—H9A	108.6
O3^viii^—K2—C10^ix^	101.75 (4)	C10—C9—H9A	108.6
O3^ix^—K2—C10^ix^	16.46 (4)	C8—C9—H9B	108.6
O1^vii^—K2—C10^ix^	158.61 (4)	C10—C9—H9B	108.6
O1—K2—C10^ix^	85.89 (4)	H9A—C9—H9B	107.6
O4^x^—K2—C10^ix^	108.29 (4)	O3—C10—O4	122.99 (16)
O4^iv^—K2—C10^ix^	71.16 (4)	O3—C10—C9	120.71 (15)
C6^vii^—K2—C10^ix^	170.09 (4)	O4—C10—C9	116.27 (14)
C6—K2—C10^ix^	67.17 (4)	O3—C10—K2^ix^	37.72 (8)
C10^viii^—K2—C10^ix^	115.09 (5)	O4—C10—K2^ix^	113.96 (10)
O3^viii^—K2—K1^vi^	45.27 (3)	C9—C10—K2^ix^	116.44 (10)
			
C2^xi^—N1—C1—C2	0.0 (2)	C4—C5—C6—O1	−18.7 (2)
C2^xi^—N1—C1—C3	−178.32 (14)	C4—C5—C6—O2	162.94 (15)
N1—C1—C2—N1^xi^	0.0 (3)	C4—C5—C6—K2	40.3 (3)
C3—C1—C2—N1^xi^	178.22 (14)	C4—C5—C6—K1	−162.96 (14)
N1—C1—C2—C7	−178.30 (15)	N1^xi^—C2—C7—S2	108.63 (15)
C3—C1—C2—C7	−0.1 (2)	C1—C2—C7—S2	−72.98 (19)
N1—C1—C3—S1	97.64 (16)	C8—S2—C7—C2	−67.34 (15)
C2—C1—C3—S1	−80.62 (18)	C7—S2—C8—C9	97.89 (15)
C4—S1—C3—C1	−65.81 (15)	S2—C8—C9—C10	174.51 (12)
C3—S1—C4—C5	−87.72 (15)	K2^ix^—O3—C10—O4	−87.24 (18)
S1—C4—C5—C6	−73.29 (18)	K1^xii^—O3—C10—O4	33.7 (2)
K1^ii^—O1—C6—O2	128.23 (16)	K2^ix^—O3—C10—C9	94.51 (17)
K2—O1—C6—O2	−35.0 (2)	K1^xii^—O3—C10—C9	−144.57 (12)
K1^ii^—O1—C6—C5	−49.9 (3)	K1^xii^—O3—C10—K2^ix^	120.92 (16)
K2—O1—C6—C5	146.80 (13)	K2^xii^—O4—C10—O3	125.1 (2)
K1^ii^—O1—C6—K2	163.2 (2)	K2^xii^—O4—C10—C9	−56.6 (3)
K1^ii^—O1—C6—K1	74.9 (2)	K2^xii^—O4—C10—K2^ix^	83.1 (3)
K2—O1—C6—K1	−88.34 (15)	C8—C9—C10—O3	−177.68 (16)
K1—O2—C6—O1	−121.20 (15)	C8—C9—C10—O4	3.9 (2)
K1—O2—C6—C5	57.09 (17)	C8—C9—C10—K2^ix^	−134.75 (13)
K1—O2—C6—K2	−146.73 (7)		

Symmetry codes: (i) *x*, −*y*+2, *z*+1/2; (ii) −*x*, −*y*+2, −*z*+1; (iii) −*x*, *y*, −*z*+3/2; (iv) *x*−1/2, *y*−1/2, *z*; (v) −*x*+1/2, *y*−1/2, −*z*+3/2; (vi) −*x*, −*y*+1, −*z*+1; (vii) −*x*, *y*, −*z*+1/2; (viii) *x*−1/2, −*y*+3/2, *z*−1/2; (ix) −*x*+1/2, −*y*+3/2, −*z*+1; (x) −*x*+1/2, *y*−1/2, −*z*+1/2; (xi) −*x*+1/2, −*y*+5/2, −*z*+1; (xii) *x*+1/2, *y*+1/2, *z*.

### Poly[(µ-3,3'-{[(3,6-bis{[(2-carboxyethyl)sulfanyl]methyl}pyrazine-2,5-diyl)bis(methylene)]bis(sulfanediyl)}dipropionato)dipotassium] (K2H2L1) Hydrogen-bond geometry (Å, º)

**Table:**

*D*—H···*A*	*D*—H	H···*A*	*D*···*A*	*D*—H···*A*
O4—H4*O*···O2^xii^	0.85 (2)	1.61 (2)	2.4637 (16)	177 (3)
C4—H4*A*···N1	0.99	2.44	3.266 (2)	141
C8—H8*A*···O3^xiii^	0.99	2.53	3.436 (2)	151

Symmetry codes: (xii) *x*+1/2, *y*+1/2, *z*; (xiii) *x*, −*y*+2, *z*−1/2.
